# 8-Oxoguanine DNA Glycosylase1 conceals oxidized guanine in nucleoprotein-associated RNA of respiratory syncytial virus

**DOI:** 10.1371/journal.ppat.1012616

**Published:** 2024-10-16

**Authors:** Lang Pan, Ke Wang, Wenjing Hao, Yaoyao Xue, Xu Zheng, Ritwika S. Basu, Tapas K. Hazra, Azharul Islam, Yashoda Hosakote, Bing Tian, Matthieu G. Gagnon, Xueqing Ba, Istvan Boldogh

**Affiliations:** 1 Department of Microbiology and Immunology, University of Texas Medical Branch at Galveston, Galveston, Texas, United States of America; 2 Institute of Genetics and Developmental Biology, Chinese Academy of Sciences, Beijing, China; 3 Department of Internal Medicine, University of Texas Medical Branch at Galveston, Galveston, Texas, United States of America; 4 Department of Biochemistry and Molecular Biology, University of Texas Medical Branch at Galveston, Galveston, Texas, United States of America; 5 Sealy Center for Structural Biology and Molecular Biophysics, University of Texas Medical Branch at Galveston, Galveston, Texas, United States of America; 6 Key Laboratory of Molecular Epigenetics of Ministry of Education, School of Life Science, Northeast Normal University, Changchun, Jilin, China; Boston University, UNITED STATES OF AMERICA

## Abstract

Respiratory syncytial virus (RSV), along with other prominent respiratory RNA viruses such as influenza and SARS-CoV-2, significantly contributes to the global incidence of respiratory tract infections. These pathogens induce the production of reactive oxygen species (ROS), which play a crucial role in the onset and progression of respiratory diseases. However, the mechanisms by which viral RNA manages ROS-induced base oxidation remain poorly understood. Here, we reveal that 8-oxo-7,8-dihydroguanine (8-oxoGua) is not merely an incidental byproduct of ROS activity but serves as a strategic adaptation of RSV RNA to maintain genetic fidelity by hijacking the 8-oxoguanine DNA glycosylase 1 (OGG1). Through RNA immunoprecipitation and next-generation sequencing, we discovered that OGG1 binding sites are predominantly found in the RSV antigenome, especially within guanine-rich sequences. Further investigation revealed that viral ribonucleoprotein complexes specifically exploit OGG1. Importantly, inhibiting OGG1’s ability to recognize 8-oxoGua significantly decreases RSV progeny production. Our results underscore the viral replication machinery’s adaptation to oxidative challenges, suggesting that inhibiting OGG1’s reading function could be a novel strategy for antiviral intervention.

## Author summary

Respiratory tract infections caused by RSV present a significant public health concern, especially affecting infants and the elderly. Effective antiviral strategies are urgently needed against this non-segmented, negative-sense RNA virus of the *Pneumoviridae* family. Our research reveals how RSV maintains replication fitness despite oxidative damage, a common exacerbator of respiratory conditions. We have identified a mechanism where RSV preserves its genome integrity under oxidative stress using the DNA repair enzyme OGG1. This enzyme ensures that the viral RNA accurately pairs 8-oxoGua with cytosine, crucial for maintaining the fidelity of the viral genome. Targeting the interplay between OGG1 and RSV RNA offers a promising approach for developing novel antiviral treatments, potentially countering the virus’s evolutionary adaptations.

## Introduction

Infections by respiratory viruses constitute a global health challenge, impacting a broad demographic range including neonates, the elderly, and individuals with underlying health conditions. The spectrum of these pathogens encompasses, but is not limited to, the respiratory syncytial virus (RSV), Sendai virus, influenza viruses, and human coronaviruses. These agents target the respiratory tract, both upper and lower segments. In the latter, infections can escalate in severity, leading to bronchiolitis, pneumonia, and severe acute respiratory syndrome [1]. A shared feature of these infections is the increased production of reactive oxygen species (ROS), which disrupts cellular redox balance, damages macromolecules, and alters physiological pathways [2–7]. These effects collectively can lead to a pathological state within the virus-infected cells and organs. Although the relationship between excessive ROS production and disease severity is recognized, the impact of ROS-induced modifications on viral macromolecules, especially RNA, warrants further investigation.

RNAs are significantly more prone to oxidative modifications than DNA [8], a difference largely due to their single-stranded nature, limited or absent protective protein associations, and proximity to oxidoreductase sites, which are sources of ROS. Among nucleic acid bases, guanine (Gua) in RNA (as well as in DNA and guanine nucleoside triphosphate pools) is especially susceptible to ROS attack, primarily due to its lowest oxidation potential [9]. The primary oxidative modification of Gua identified in cellular RNA is 8-oxo-7,8-dihydroguanine (8-oxoGua) [10]. While 8-oxoGua serves as an established indicator of oxidative damage in nucleic acids, the detailed mechanisms by which it impacts viral RNA, along with its broader implications for the management of viral infections, remain to be thoroughly elucidated. Furthermore, virus replication entails a sophisticated integration into the host cells (or virocells) [11], where the virus not only triggers ROS production but also induces DNA damage [12,13]. Direct evidence of DNA damage at the onset of RSV infection, demonstrated by comet assays [14], underscores the intricate relationship between viral replication and the host’s oxidative stress response. This indicates a shared strategy among viruses to evade host damage detection mechanisms and achieve successful infection, a tactic observed in RNA viruses such as the Hendra virus, Nipah virus, and SARS-CoV-2 [15,16].

Previous research has underscored the significant role of ROS, induced by RSV, in activating gene promoter function. This activity significantly alters cellular gene-expression signatures [17–20] and triggers activation of DNA damage response signaling pathways [21,22], promoting the expression of pro-inflammatory and antiviral genes that contribute to lung pathogenesis. Central to mitigating oxidative damage, 8-oxoguanine DNA glycosylase 1 (OGG1) plays a crucial role in identifying 8-oxoGua and initiating the base excision repair (BER) pathway, which is an essential cellular defense mechanism against oxidative stress [23,24]. While traditionally associated with mutagenesis, recent insights suggest that 8-oxoGua also acts as an epigenetic-like marker, particularly evident in pulmonary disease contexts involving OGG1 [25,26]. This interaction is pivotal in the recruitment of transcription factors like NF-κB and SMAD3 to their specific binding sites [27–31], thereby enhancing gene expression crucial for innate immune responses to viral infections. However, further exploration is needed to fully understand OGG1’s specific role in identifying 8-oxoGua within RSV RNA and its subsequent effects on the interplay between the virus and its host.

In this study, we highlight the selective accumulation of 8-oxoGua within the RSV nucleoprotein (N)-associated RNAs, supported by LC_MS/MS and OGG1 RNA-immunoprecipitation followed by sequencing. Through co-precipitation assays and mass spectrometry, we identified the N protein as a principal binding partner of OGG1. We observed a significant reduction in RSV progeny production upon impairment of OGG1’s ability to detect 8-oxoGua, highlighting OGG1’s critical, albeit transient, role in N-RNA complex association. This discovery opens new avenues for understanding how RSV, and potentially other respiratory RNA viruses, tolerate guanine oxidation to enhance genome fidelity. Furthermore, our findings underscore the potential of OGG1 inhibitors as an innovative approach to lower viral replication precision, presenting a promising avenue for antiviral development.

## Results

### Oxidative stress plays a crucial role in enhancing RSV progeny production

**Fig 1:**
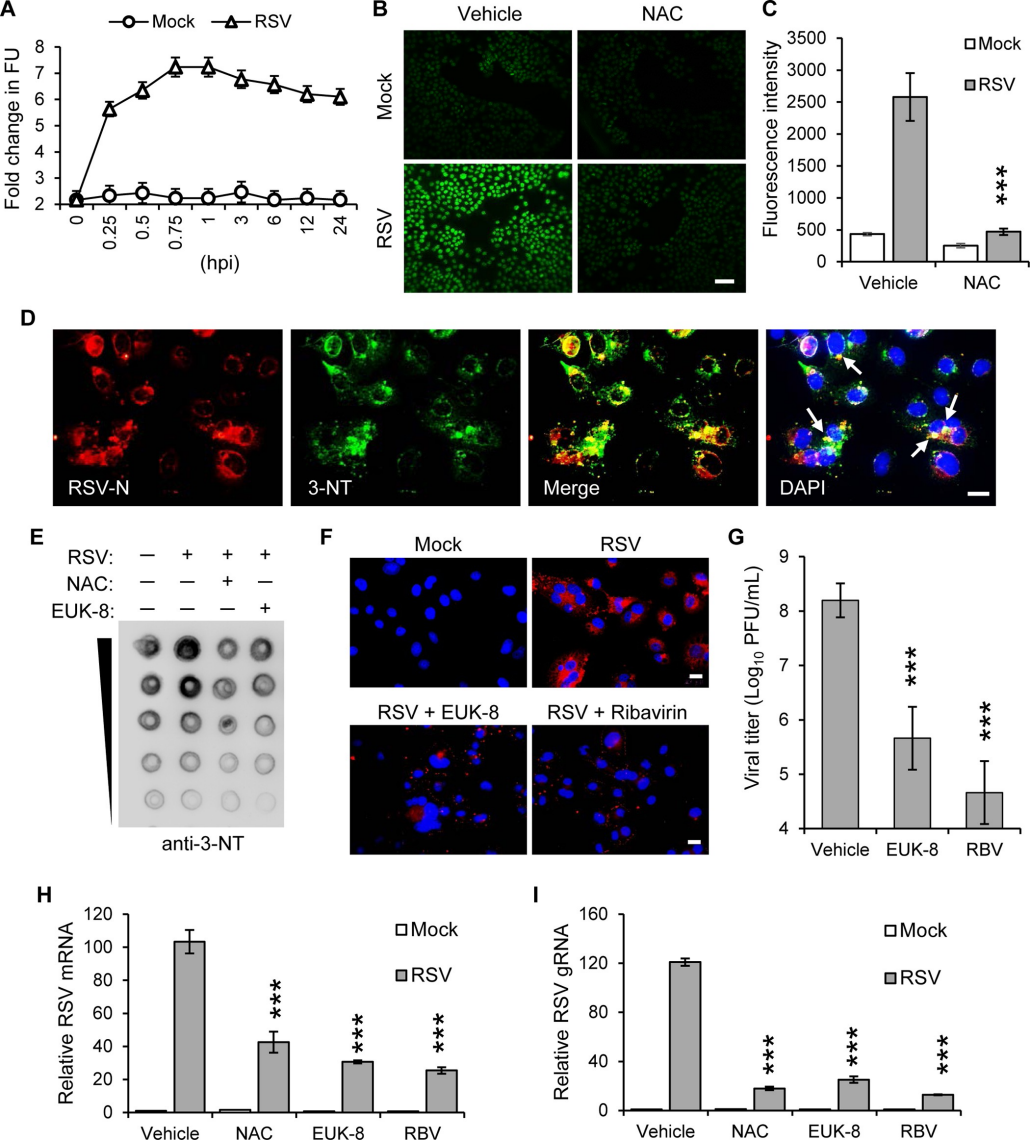
The necessity of oxidative stress for the RSV lifecycle. (A) Kinetic changes in ROS levels were measured by 2’, 7’-dichlorofluorescein (DCF) in hSAECs infected with RSV (MOI = 1). (B) Detection of ROS levels using CellROX Green with imaging (MOI = 1, 2 hpi). Treatment with NAC (10 mM) was initiated post inoculum. Scale bar, 100 μm. (C) Quantification of fluorescence intensity from the ROS sensor using a microplate reader at an excitation/emission of 485/520 nm. n = 4 biological replicates. (D) Immunostaining of RSV N protein as well as ROS-induced protein Nitrotyrosine (3-NT) after RSV infection (MOI = 1, 24 hpi). Nuclear DNA was stained with DAPI. White arrows indicate the colocalization of RSV N and proteins carrying 3-NT modifications. Scale bar, 20 μm. (E) A representative dot-blot image showing global 3-NT levels in proteins (MOI = 1, 24 hpi). Treatments with NAC (10 mM) or EUK-8 (500 μM) were initiated post inoculum. (F) Immunostaining of RSV M2-1 protein at 24 hpi, MOI = 1. Treatments with EUK-8 (500 μM), and Ribavirin (20 μM) were initiated post inoculum. Blue fluorescence, DAPI staining. Displayed images are representative of more than 20 fields of view, encompassing over 100 cells from two separate experiments. Scale bar, 20 μm. (G) Viral titers were evaluated 3 days post-infection using a plaque assay in hSAECs infected with RSV (MOI = 0.1). Treatments with vehicle (addition of solution without inhibitor), EUK-8 (500 μM), and Ribavirin (20 μM) were initiated post inoculum. n = 4. (H-I) hSAECs were infected with RSV (MOI = 1), treatment with NAC (10 mM), EUK-8 (100 μM) or Ribavirin (20 μM) was initiated post inoculum. Total RNA was extracted, and mRNA was selected by Oligo dT beads. Levels of RSV *G* mRNA (H) and gRNA (I) were determined by qRT-PCR. Information on primers sequences is provided in S1 Table. Values are presented as mean ± SD. n = 3. ***p < 0.001 by unpaired Student’s *t* test.

Given the tropism of RSV to the respiratory epithelium [32], we utilized human small airway epithelial cells (hSAECs) in our study. To examine the impact of oxidative stress on RSV infection, we initially measured the levels of ROS in our cell model post-infection (Fig 1A). Using fluorogenic probes for dynamic live cell imaging, we observed a significant increase in ROS following RSV infection, an elevation that was effectively reduced by treatment with N-acetyl-L-cysteine (NAC) (Fig 1B and 1C). Our analysis further indicated that RSV N proteins experienced nitrosylation modifications (Fig 1D), suggesting oxidative modifications affecting viral components. We then utilized EUK-8, a salen-manganese complex known for emulating the antioxidant functions of superoxide dismutase, catalase, and glutathione peroxidase [33]. EUK-8 significantly attenuated oxidative stress markers, including protein nitrosylation triggered by RSV (Fig 1E), demonstrating its potential in reducing oxidative modifications. A comparative analysis with Ribavirin, an established antiviral agent against RSV [34], revealed that both EUK-8 and Ribavirin markedly decreased levels of the M2-1 protein (Fig 1F), suggesting their efficacy in disrupting viral production. Importantly, treatment with EUK-8 or Ribavirin led to a notable decrease in infectious RSV progeny yield (Fig 1G) and a reduction in viral RNA levels (Fig 1H and 1I). These results collectively highlight the critical effect of ROS on RSV mRNA and genome (g) RNA abundance.

### 8-oxoGua manifests as an acquired phenotype within the N protein-associated RNA

**Fig 2:**
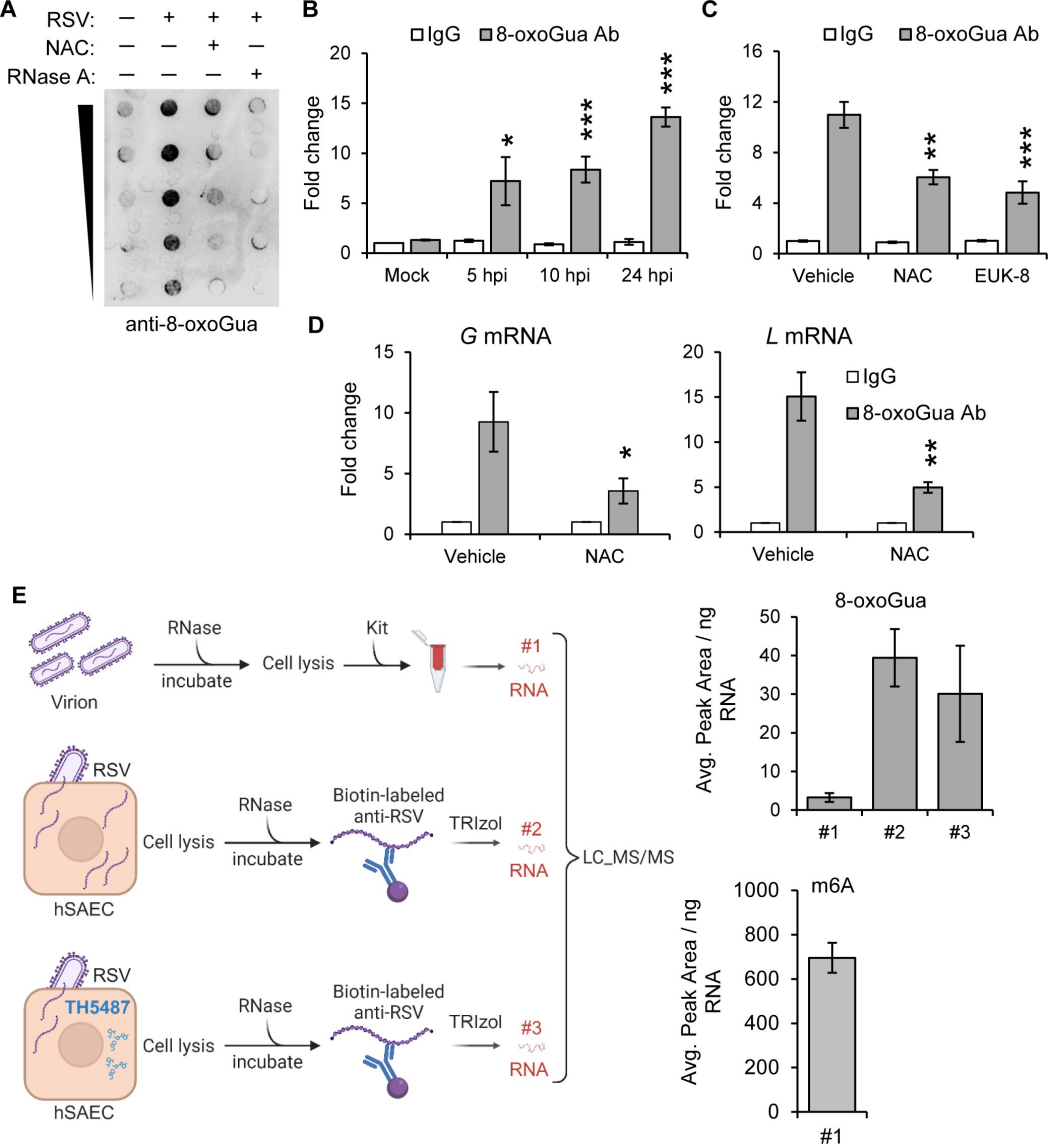
Acquisition of 8-oxoGua in RSV RNA within virocells. (A) Representative RNA dot-blot illustrating 8-oxoGua levels. Total RNA from virocells (MOI = 1, 24 hpi) or treatment with NAC (10 mM) was serially diluted twofold and probed with an anti-8-oxoGua antibody. (B) Anti-8-oxoGua antibody pull down from total RNA extracted from virocells (MOI = 1). Levels of RSV gRNA in immunoprecipitants were quantified by qRT-PCR. (C) Immunoprecipitation using anti-8-oxoGua antibody from total RNA extracted from virocells (MOI = 1, 24 hpi), with treatments of NAC (10 mM) or EUK-8 (100 μM) initiated post-inoculum. Levels of RSV gRNA in immunoprecipitants were determined by qRT-PCR. (D) Quantification of 8-oxoGua levels in RSV G and L mRNA by qRT-PCR. Total RNA was extracted from virocells (MOI = 1, 24 hpi), mRNA was enriched using Oligo dT beads, and incubated with 8-oxoGua or negative control antibody. In B-D, Fold change was calculated by 2^- [Ct (test Ab)-Ct (negative Ab)]. (E) Quantification of 8-oxoGua and m6A levels by LC_MS/MS. Left panel: Strategy for extracting viral RNA from RSV virions or virocells (MOI = 1, 24 hpi). Treatment with TH5487 (10 μM) was initiated post inoculum. RSV virions were purified by sucrose ultracentrifugation from cell culture supernatant. Biotin-labeled RSV antibody was used to pull down N protein-associated RNA from virocells without crosslinking. Right panel: Graphical representation of 8-oxoGua level in virions (#1) and N protein-associated RNA from virocells (#2 and #3, upper right panel). m6A levels were exclusively assessed in virions (#1, lower right panel). Data are expressed as the average peak area per ng RNA. n = 3 biological replicates. Statistical analysis was conducted using an unpaired Student’s t test, with results shown as means ± SD. Significance levels are indicated as *p<0.05, **p < 0.01, ***p < 0.001. Fig 2E created with Biorender.com.

Following RSV infection, our analysis of total RNA revealed a significant increase in 8-oxoGua levels, a rise that was effectively reduced by treatment with NAC (Fig 2A). This finding aligns with RNA’s inherent susceptibility to ROS and guanine’s particular vulnerability due to its low oxidation potential, predisposing it to heightened 8-oxoGua levels under oxidative stress. To specifically identify 8-oxoGua within viral RNA, we employed an 8-oxoGua-specific antibody for RNA immunoprecipitation (IP) from cells infected with RSV. Quantitative RT-PCR (qRT-PCR) analysis thereafter demonstrated a significant enrichment of RSV gRNA by the anti-8-oxoGua antibody compared to a control immunoglobulin G (IgG) (Fig 2B). Additionally, treatments with NAC and EUK-8 led to a decrease in viral gRNA levels (Fig 2C), indicating these as effective methods for counteracting oxidative alterations in viral RNA. To investigate the presence of 8-oxoGua in RSV mRNA, we first isolated mRNA from the total RNA sample using Oligo dT, followed by IP with the anti-8-oxoGua antibody. qRT-PCR analysis revealed a significant enrichment of the *G* and *L* mRNA with 8-oxoGua, a phenomenon mitigated by NAC treatment (Fig 2D), thereby confirming that RSV mRNA undergoes modification by 8-oxoGua.

Given the protective effect of N protein against degradation of RSV RNA [35], we treated virocells lysates with RNase to eliminate unprotected RNA, subsequently isolating N protein-associated RNA using a Biotin-labeled anti-RSV antibody. The IP experiment confirmed the presence of the N protein in the pull-down (S1 Fig). The use of liquid chromatography-electrospray ionization tandem mass spectrometry (LC_MS/MS) to quantify 8-oxoGua in the isolated RNA revealed a notable oxidative modification extent of 0.4% ± 0.08 of guanine bases in nucleocapsid-associated RNA from virocells, while this modification was nearly absent in RNA from virions (Fig 2E). This finding highlights the predominance of oxidative modifications within host cell environment. Additionally, application of TH5487, a selective inhibitor of OGG1’s active site [36], did not markedly alter the levels of 8-oxoGua (Fig 2E), suggesting that inhibition of OGG1 by TH5487 insignificantly affects 8-oxoGua levels in RNAs associated with the N protein from virocells. As observed, we detected low levels of 8-oxoGua in virions, which prompted us to investigate whether this observation was due to technical limitations in our assay system. Consequently, we included N6-methyladenosine (m6A) as an additional marker, given its well-established presence in virion RNA. The identification of m6A at an amount of 0.1% ± 0.02 of adenosines in virions (Fig 2E) is consistent with previous findings [37]. These results provide insight into the acquisition of 8-oxoGua in RNAs interacting with the N protein because of RNA vulnerability to ROS, highlighting the intricate relationship between viral adaptation and the host cellular environment.

### OGG1 reads 8-oxoGua in RNA without glycosylase activity

**Fig 3:**
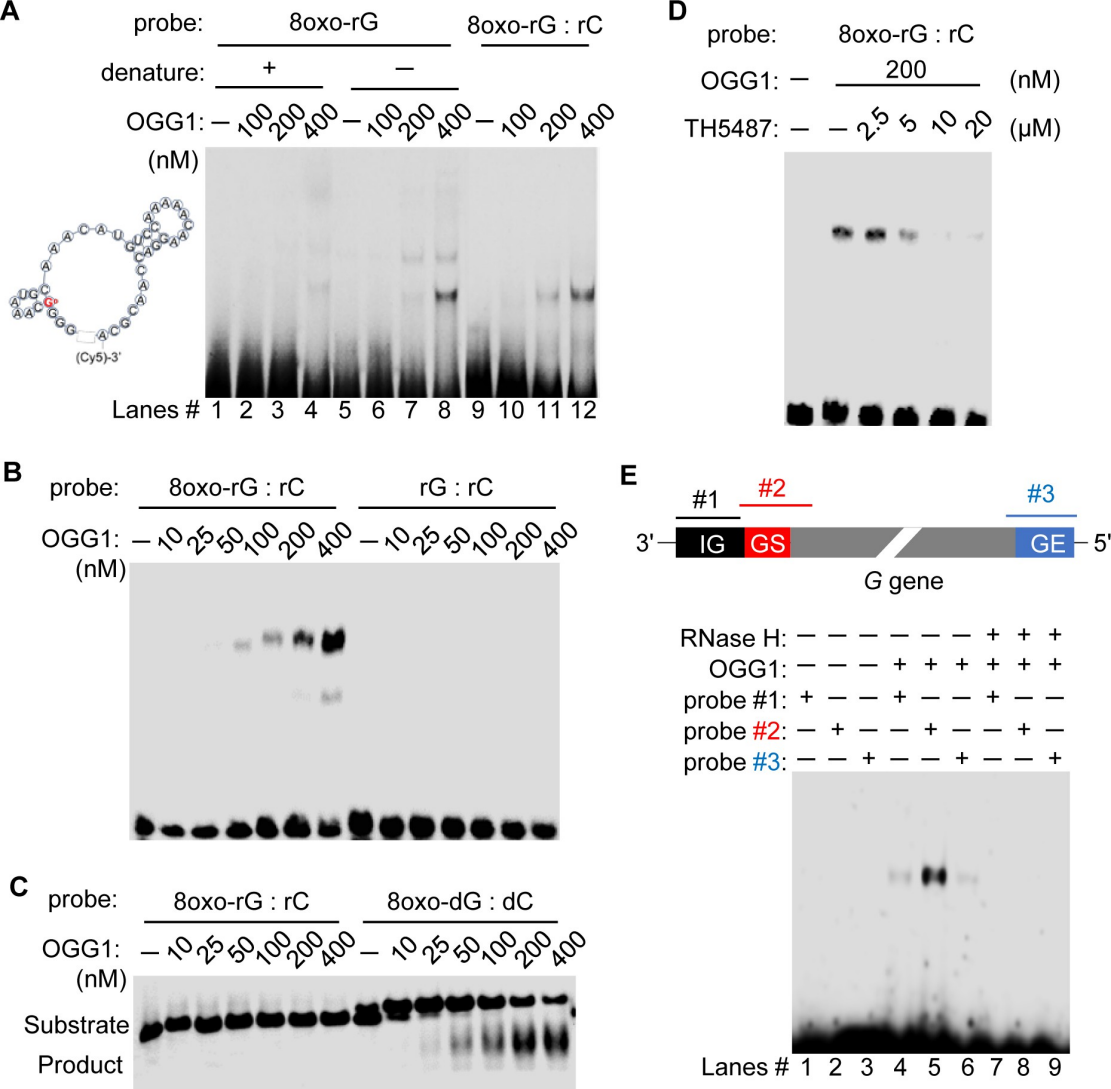
OGG1 recognizes 8-oxoGua in RNA. (A) OGG1 binds RNA containing 8-oxoGua. The secondary structure of the undenatured single-strand probe was predicted using software as described in Materials and Methods. The probe contains a single 8-oxoGua (marked in red) at the end of GGGG. EMSA was performed using single-stranded RNA (8oxo-rG), either denatured at 95°C for 5 min (Lanes 1–4) or undenatured (Lanes 5–8). Additionally, EMSA was conducted with the double-stranded RNA probe (8oxo-rG: rC), where 8oxo-rG anneals with its complementary genome sequence (rC) (Lanes 9–12). (B) Representative EMSA image demonstrating OGG1’s binding affinity for annealed RNA containing 8-oxoGua. (C) Representative image showing OGG1’s excision activity on 8-oxoGua within DNA, contrasting with its inactivity towards RNA. (D) EMSA visualization indicating that TH5487 impedes OGG1’s binding to RNA harboring 8-oxoGua. (E) EMSA depiction showing OGG1’s interaction with a DNA-RNA hybrid that includes the gene-start (GS) motif. Total RNA (1 μg) extracted from virocells (MOI = 1, 24 hpi) was hybridized with Cy5-labelled DNA probes (20 nM) containing the intergenic region (IG), the gene-start (GS), and the gene-end (GE) of G gene. Following incubation with recombinant OGG1 (100 nM), the assay proceeded to EMSA. Oligo sequences for EMSA and OGG1 glycosylase activity are listed in S2 Table. n = 3 biological replicates.

To explore OGG1’s direct engagement with RNA containing 8-oxoGua, we synthesized RNA oligonucleotides mirroring sequences from the RSV antigenome. Electrophoretic mobility shift assays (EMSA) revealed that heating the RNA to denature its secondary structures abolished OGG1’s ability to bind (Fig 3A, lanes 1–4) while OGG1 retained its binding affinity to non-denatured RNA probes containing 8-oxoGua (Fig 3A, lanes 5–8). This suggests that OGG1’s interaction with single-stranded RNA relies on temperature-sensitive secondary structures. Given the requirement of complementary cytosine for OGG1 to recognize 8-oxoGua [38], we also tested OGG1 with double-stranded RNA containing 8-oxoGua (Fig 3A, lanes 9–12), observing similar binding preferences as with non-denatured single-stranded RNA. Further comparisons with RNA lacking 8-oxoGua confirmed OGG1’s selective affinity for RNA oligonucleotides that include 8-oxoGua (Fig 3B), thereby reinforcing the critical role of 8-oxoGua for OGG1’s interaction with RNA.

Despite OGG1’s well-documented DNA glycosylase function in removing 8-oxoGua from DNA, we investigated its effect on oxidized RNA. Our experiments demonstrated that while OGG1 exhibits significant glycosylase activity towards DNA containing 8-oxoGua, it lacks similar activity on RNA containing 8-oxoGua, even at higher enzyme concentrations (Fig 3C). This specificity points to a unique mode of action in RNA interactions. Moreover, we observed that TH5487 blocks OGG1’s binding to RNA with 8-oxoGua in a dose-dependent manner (Fig 3D), suggesting that TH5487 could disrupt the OGG1-RNA interaction. To further examine whether the active site of OGG1 mediates its binding to RNA containing 8-oxoGua, we employed both wild-type OGG1 and OGG1 mutants. The phenylalanine 319 alanine (F319A) mutant exhibits weak affinity for 8-oxoGua and lacks base excision activity [39], whereas the lysine 249 glycine mutant (K249Q) retains the ability to bind 8-oxoGua but lacks base excision activity [40]. Our results showed that the OGG1 F319A mutant did not bind to the RNA oligonucleotides containing 8-oxoGua (S2 Fig). In contrast, the K249Q mutant exhibited increased binding to RNA with 8-oxoGua, confirming the specificity and importance of OGG1’s active site in mediating this interaction. Furthermore, to determine OGG1’s binding preferences within viral RNA, we hybridized synthetic DNA probes (S2 Table) containing the *cis*-acting intergenic (IG), gene-start (GS), and gene-end (GE) signals of the G gene with total RNA extracted from virocells. Our hybridization followed by EMSA revealed OGG1’s pronounced preference for the GS-containing probe-RNA hybrid (Fig 3E). This preference suggests that the complementary GS region in the antigenome may contain more OGG1 substrates compared to the complimentary IG and GE regions. Applying RNase H, which targets DNA-RNA hybrids for degradation, effectively eliminated this binding, underscoring the binding specificity of OGG1 for RNA as part of double-stranded structures.

**Fig 4:**
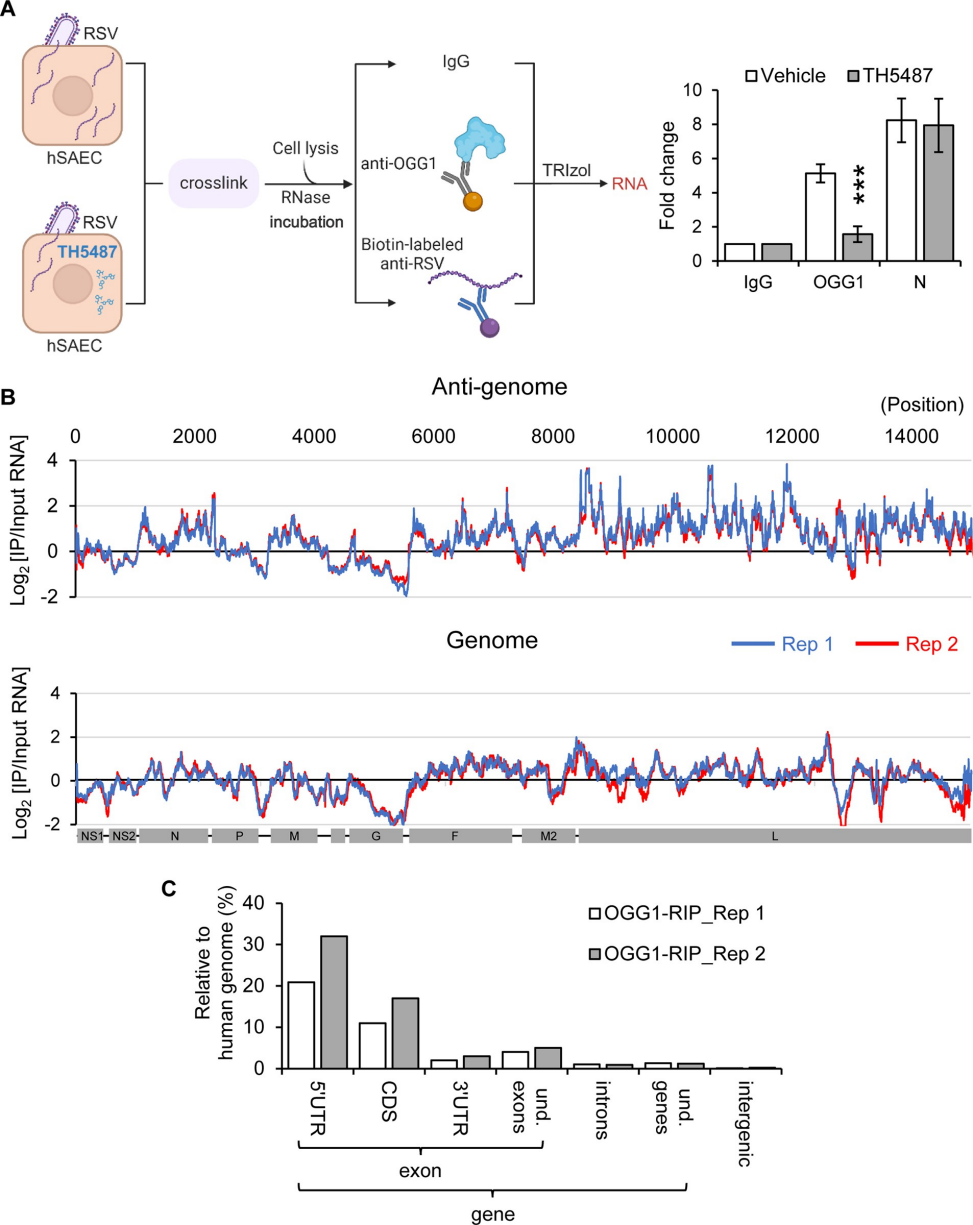
OGG1 binding landscape across the viral genome. (A) Left panel: Strategy for enrichment of viral RNAs interacting with OGG1 and N protein from virocells after formaldehyde crosslinking. Treatment with TH5487 (10 μM) was initiated post inoculum (MOI = 1, 24 hpi). RNA-immunoprecipitation (RIP) was carried out using antibodies against OGG1 or RSV N protein. Right panel: Quantification of RSV genomic RNA levels after antibody pull-down, determined by qRT-PCR. Fold change was calculated by 2^- [Ct (test Ab)-Ct (negative Ab)]. n = 3 biological replicates. Statistical analysis was conducted using an unpaired Student’s *t* test, with results shown as means ± SD. ***p<0.001. (B) OGG1 RIP-Seq analysis in virocells (MOI = 1, 24 hpi) illustrating the distribution of RSV-specific reads across two independent experiments (visualized in blue and red tracks). Data are presented as the log2 ratio of reads obtained from anti-OGG1 immunoprecipitation relative to input RNA, mapped against the RSV genome (GenBank: M74568.1). Peaks were called positive if the log2 enrichment score was ≥1. (C) Annotation and categorization of cellular RNA reads obtained from OGG1 RIP-Seq aligned to the human genome HG38 (GenBank: GCA_000001405.29), presented as the percentage of OGG1-RIP peaks. UTR, untranslated region of mRNAs; CDS, exon regions that code for protein; und., undefined. Fig 4A created with Biorender.com.

Upon confirming that the interaction between OGG1 and RNA depends on the presence of 8-oxoGua, we isolated OGG1-RNA complexes along with viral nucleocapsid from virocells. Prior to IP, RNase pre-treatment was employed to emphasize OGG1’s association with RNAs protected by the N protein (Fig 4A, left panel). This approach revealed a marked enrichment of viral gRNA associated with both OGG1 and the N protein (Fig 4A, right panel). Notably, treatment with TH5487 led to a decrease in the detection of OGG1-enriched RNA (Fig 4A, right panel), highlighting the specificity of OGG1’s interaction with viral RNA containing 8-oxoGua.

To map the OGG1 binding sites across the viral genome comprehensively, we performed high-throughput sequencing of OGG1-enriched RNAs (Fig 4B). Data are presented as log2 of the ratio of reads recovered from the immunoprecipitated to input RNA as described previously [41]. Our analysis pinpointed specific sequences—5’-GGGGCAAAT-3’ for the NS1, N, P, M, G, F, and M2 genes, and 5’-GGGACAAAAT-3’ for the L gene within the antigenome—that coincide with OGG1-RNA IP (RIP) peaks (S3 Fig). These sequences are complementary to the highly conserved 9-nucleotide gene start signal (3’-CCCCGUUUA-5’) found at the beginning of each gene in the RSV genome [42]. Furthermore, we have identified sequences featuring 5’-GAACCT, 5’-TACGG, and 5’-GGACT, known as consensus sequences for m6A methylation [43], which overlap with OGG1 RIP-Seq peaks (S3 Fig). The crosslinked RNA also exhibited significant enrichment for cellular RNA sequences, predominantly within the 5’UTR of host RNAs (Fig 4C), suggesting that OGG1 also interacts with host RNA in addition to viral RNAs. After RNase digestion, the significant enrichment of cellular RNAs crosslinked to OGG1 in our results indicates that these host RNAs are likely protected by RNA-binding proteins. Thereby, OGG1 potentially affects host gene expression as well.

### 8-oxoGua ‘reader’ protein OGG1 hijacked by RSV nucleoprotein

**Fig 5:**
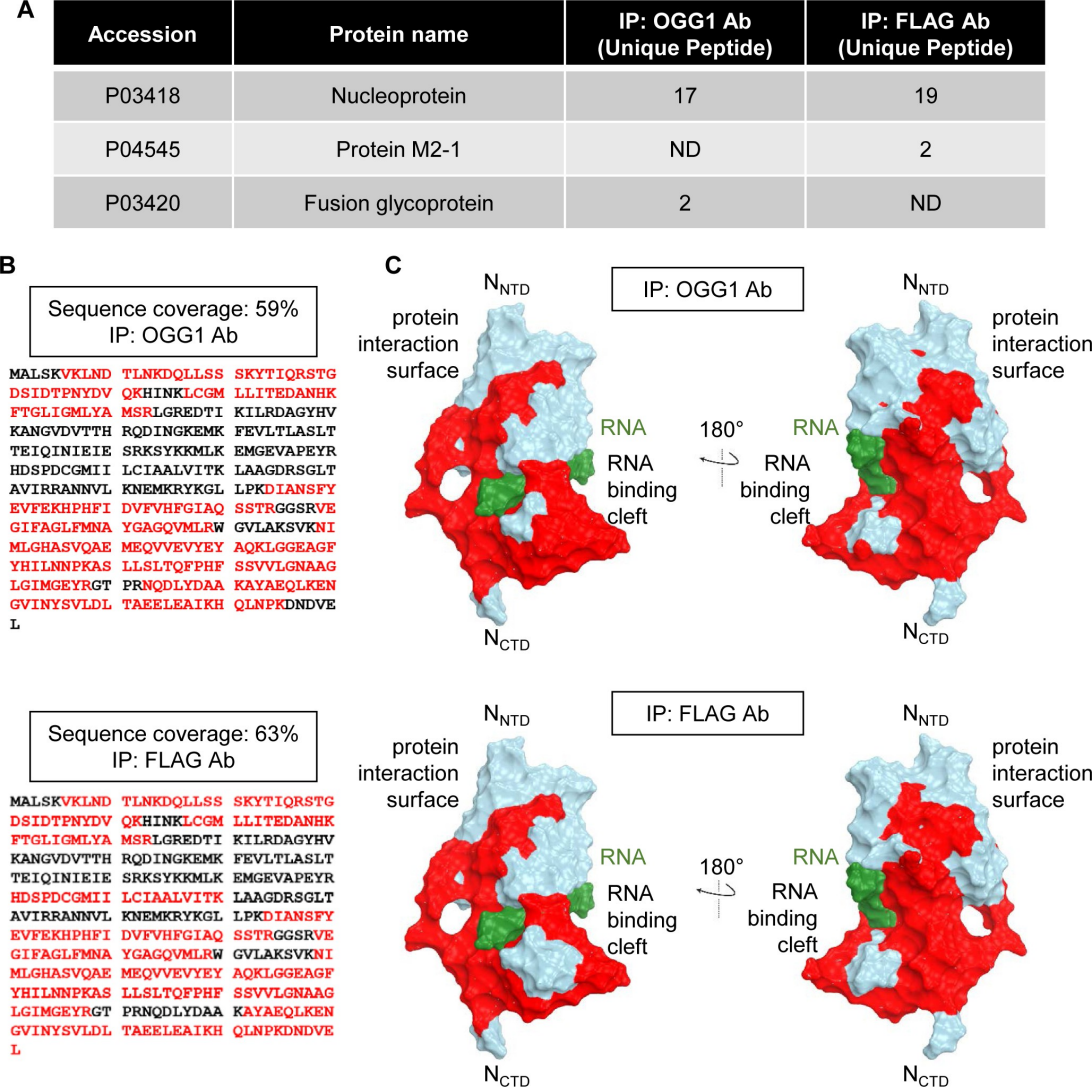
OGG1 Interactome post-RSV infection. (A) LC_MS/MS identification of RSV proteins co-immunoprecipitated with OGG1 (RSV MOI = 1, 24 hpi). The analysis was conducted on complexes isolated using an anti-OGG1 antibody (for endogenous OGG1) and anti-FLAG antibody (for transgenically expressed OGG1). Proteins are listed according to the number of significant peptides identified. ND not detected. (B) The entire amino acid sequence of the RSV N protein was visualized, with residues identified by LC_MS/MS highlighted in red. (C) The peptides identified by LC_MS/MS in red were visualized within the three-dimensional structure of the N protein, with RNA depicted in green. NTD, N terminal domain. CTD, C terminal domain.

**Fig 6:**
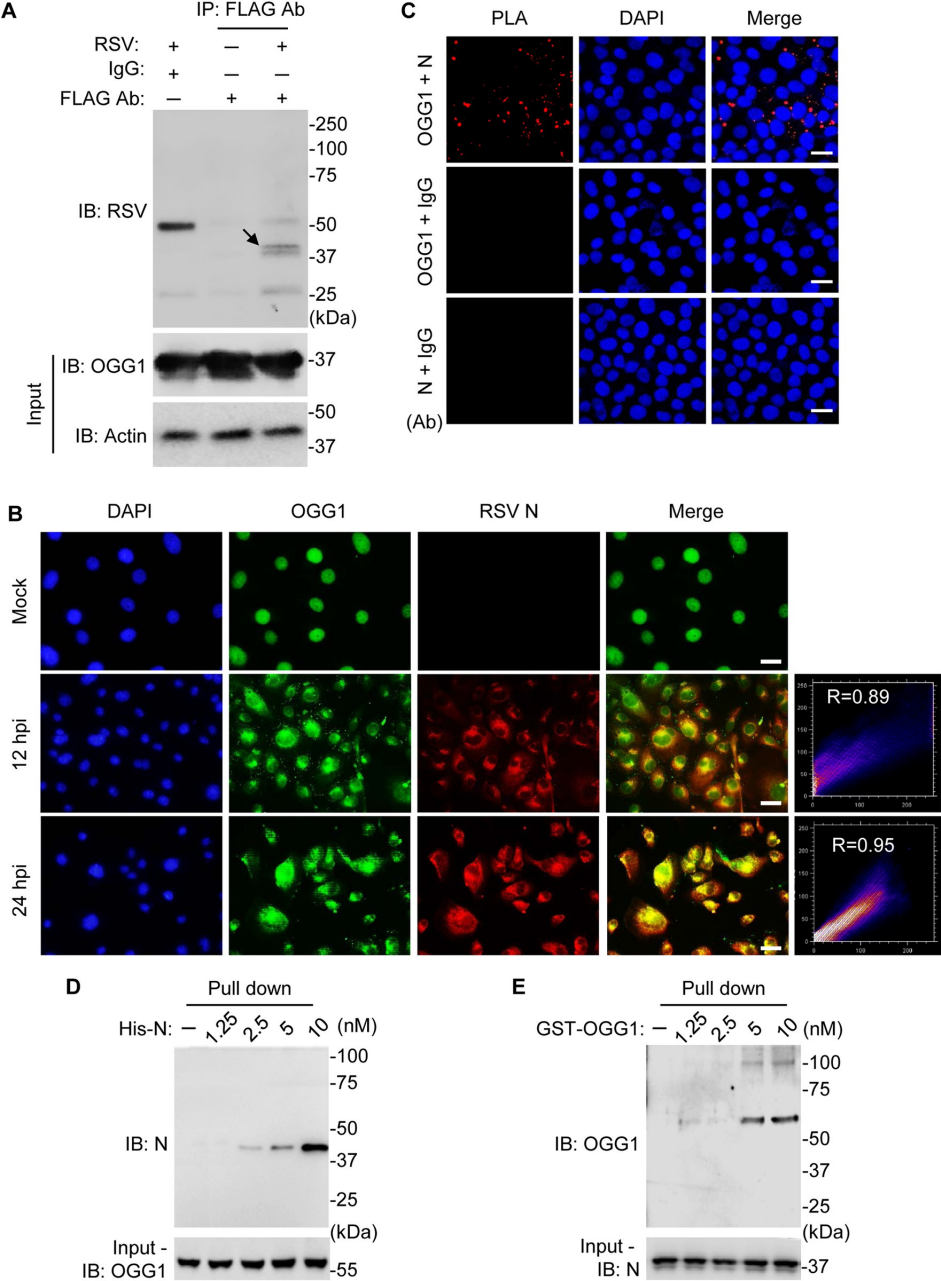
Interaction between OGG1 and RSV nucleoprotein (N). (A) Immunoblot (IB) analysis of co-immunoprecipitated extracts from Flag-OGG1 expressing hSAECs infected with RSV (MOI = 1, 24 hpi). Co-immunoprecipitation was performed using an anti-FLAG antibody or control IgG, and the blots were subsequently probed with an anti-RSV antibody. Anti-Actin immunoblotting from whole cell lysates served as a loading control. An arrow indicates the N protein. (B) Immuno-fluorescence staining of OGG1 and RSV N protein. hSAECs were mock or RSV-infected (MOI = 1), and cells were fixed at 18 and 24 hpi. The co-localization measurement between fluorophores (Alexa 594 vs. Alexa 488) was assessed using the intensity of individual fluorophore pixels, calculated as the Pearson correlation coefficient (R). Scale bars: 20 μm. (C) Proximity ligation assay (PLA) shows OGG1 and N interactions (MOI = 1, 24 hpi). PLA signals were absent when control IgG were applied. Scale bars, 50 μm. (D-E) GST pull-down assays demonstrating the interaction between OGG1 and the N protein. Immobilized GST-tagged OGG1 (100 nM) was incubated with increasing concentrations of His-tagged N, followed by washing, SDS-PAGE and immunoblotting using an anti-N antibody. (D) In a reciprocal setup, His-tagged N protein (100 nM) was incubated with varying concentrations of GST-OGG1, followed by immunoblotting with an anti-OGG1 antibody (E).

The potential involvement of OGG1 in RSV life cycle may lie in its ability to recognize 8-oxoGua within nucleocapsids and to establish connections with viral proteins. To investigate this hypothesis, hSAECs were transgenically expressing a Flag-tagged OGG1 [44], enabling the isolation of OGG1-associated complexes through IP with anti-OGG1 and anti-FLAG antibodies. Following LC_MS/MS analysis, a notable co-IP of OGG1 with various RSV proteins, particularly the N protein, was observed (S3 Table). This was evidenced by the identification of numerous unique peptides specific to the N protein. Specifically, 17 unique peptides from the N protein were identified with 59% coverage in the anti-OGG1 antibody immunoprecipitants and 19 unique peptides with 63% coverage in the anti-FLAG antibody sample (Fig 5A–5C). Importantly, while this analysis demonstrates significant peptide homology with the RSV N protein, it does not directly confirm the interaction sites between OGG1 and the N protein due to the denaturation step during peptide digestion, which disrupts native protein-protein interactions. Pathway enrichment analysis of the OGG1 interactome identified associations with RNA metabolism and biogenesis pathways (S4 Fig), which suggest a potential role of OGG1 in viral RNA synthesis during RSV infection. Further validation was obtained through immunoblotting and antibody staining, which confirmed the presence of the N protein within the OGG1 complex (Fig 6A and 6B). Proximity ligation assays using OGG1 and N antibodies, rather than control IgG (Fig 6C), verified the vicinity between OGG1 and the N protein. Additionally, glutathione S-transferase (GST) pull-down assays provided direct evidence of the interaction between OGG1 and the N protein (Fig 6D and 6E), highlighting that this interaction occurs independently of the viral RNA. The colocalization of OGG1 with the M2-1 protein was further confirmed through immunostaining (S5A Fig), and direct interaction was demonstrated by GST pull-down assays (S5B and S5C Fig). Collectively, these results underscore that OGG1 not only identifies 8-oxoGua within the viral RNA but also directly interacts with viral proteins, notably the N protein.

### OGG1 loss of function decreases yields of RSV progeny

**Fig 7:**
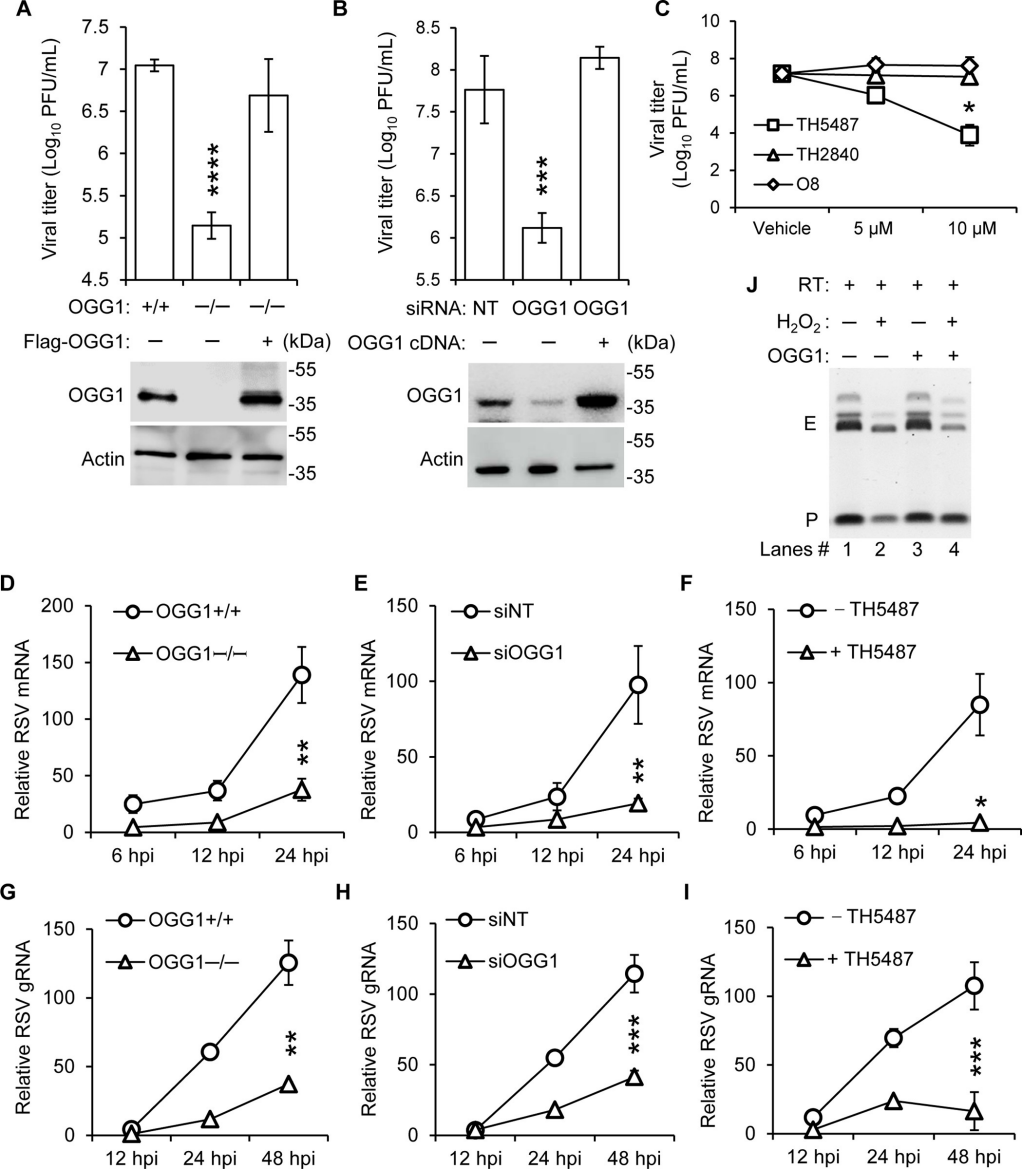
OGG1’s positive influence on the production of RSV RNA. (A-B) hSAECs were infected with RSV (MOI = 0.1) and viral titers were assessed 3 days post-infection in scenarios where OGG1 expression was either (A) down-regulated via siRNA or (B) completely eliminated by CRISPR/Cas9 knockout. NT, non-targeting siRNA. The knockout was overcompensated by ectopic expression of OGG1. The lower panel displays immunoblot analysis of OGG1 expression in cell lysates, with Actin serving as a loading control. (C) Following 1-hour post inoculation (MOI = 0.1), hSAECs were treated with TH5487, TH2840, or O8, and viral titers were determined by plaque assays (3 dpi). In D-I, hSAECs were infected with RSV (MOI = 0.5), and total RNA was extracted first at indicated times post-infection. Experiments include OGG1 knockout by CRISPR/Cas9 in hSAECs (D and G), down regulation of OGG1 expression by siRNA (E and H), and inhibition of OGG1’s reading function by TH5487 (F and I). (D-F) mRNA was isolated using Oligo dT beads, and *G* mRNA level was quantified by RT-qPCR. (G-I) After mRNA isolation, RSV gRNA levels were determined via RT-qPCR. n = 3. Statistical analysis was conducted using an unpaired Student’s *t* test, with results shown as means ± SD. Significance levels are indicated as *p<0.05, **p < 0.01, ***p < 0.001. (J) Efficiency of primer extension on RNA template containing 8-oxoGua. Moloney murine leukemia virus reverse transcriptase (RT) was used in the assay. H_2_O_2_, 800 μM. OGG1, 50 nM. P indicates primer. E indicates extension.

Silencing its mRNA or genomic deletion of OGG1 resulted in a significant reduction in RSV progeny (Fig 7A and 7B). Moreover, treatment with TH5487 consistently decreased RSV titers, underscoring the specific role of OGG1 in RSV progeny production. Control experiments using TH2840, an inactive analog of TH5487 [36], and O8, a compound known to interfere with the β-lyase activity of OGG1 [45], did not show inhibitory effects on RSV infection (Fig 7C). This underscores the unique impact of disrupting the OGG1-RNA interaction on viral yields, an effect not seen with other DNA repair enzymes such as NEIL2 or MTH1 (S6A Fig). The restoration of OGG1 expression correlated with increased viral titers, highlighting the enzyme’s essential role in RSV infection (Fig 7B). This was supported by qRT-PCR analysis of RNA from infected cells, showing that OGG1 markedly enhances both the RSV gRNA and mRNA abundance (Fig 7D–7I). Further immunoblot analysis confirmed that blocking OGG1’s ability to ‘read’ 8-oxoGua with TH5487 decreases the levels of key RSV proteins, N and M2-1 (S6B Fig), directly linking OGG1’s activity to the production of viral proteins. Additionally, multicycle growth assays demonstrated that TH5487 treatment resulted in a significant reduction in cytopathic effects, as evidenced by increased crystal violet staining (S6C and S6D Fig). To evaluate whether decreases in RSV yield were associated with cytotoxicity of OGG1 inhibitors, we assessed the extent of plasma membrane damage following RSV infection using lactate dehydrogenase (LDH) assays. The results showed that TH5487 significantly reduced the level of LDH released into the medium (S7 Fig), indicating the absence of toxic effects from TH5487. To explore the mechanistic role of OGG1 on viral RNA production, we conducted primer extension assays with reverse transcriptase (RT) on RNA templates containing 8-oxoGua. We observed that RT could efficiently bypass 8-oxoGua, synthesizing cDNA effectively (Fig 7J, lanes 1 and 3). Under oxidative stress conditions induced by the addition of H_2_O_2_, OGG1 demonstrated a protective effect, enhancing the generation of extension products (Fig 7J, lane 4 versus lane 2). Previous studies have shown that TH5487 treatment in the viral infection model significantly decreases RSV-induced inflammation, eases clinical symptoms and weight loss, and enhances antiviral immune responses [18,19]. In this study, we assessed viral titer and RNA levels in RSV-infected mice, discovering that TH5487 treatment markedly reduced virus yields (S8A Fig) and RNA levels in the lung (S8B and S8C Fig). Collectively, these findings illuminate OGG1’s role in enhancing RSV RNA abundance and suggest that targeting its reading function presents a promising avenue for developing novel antiviral strategies.

## Discussion

**Fig 8:**
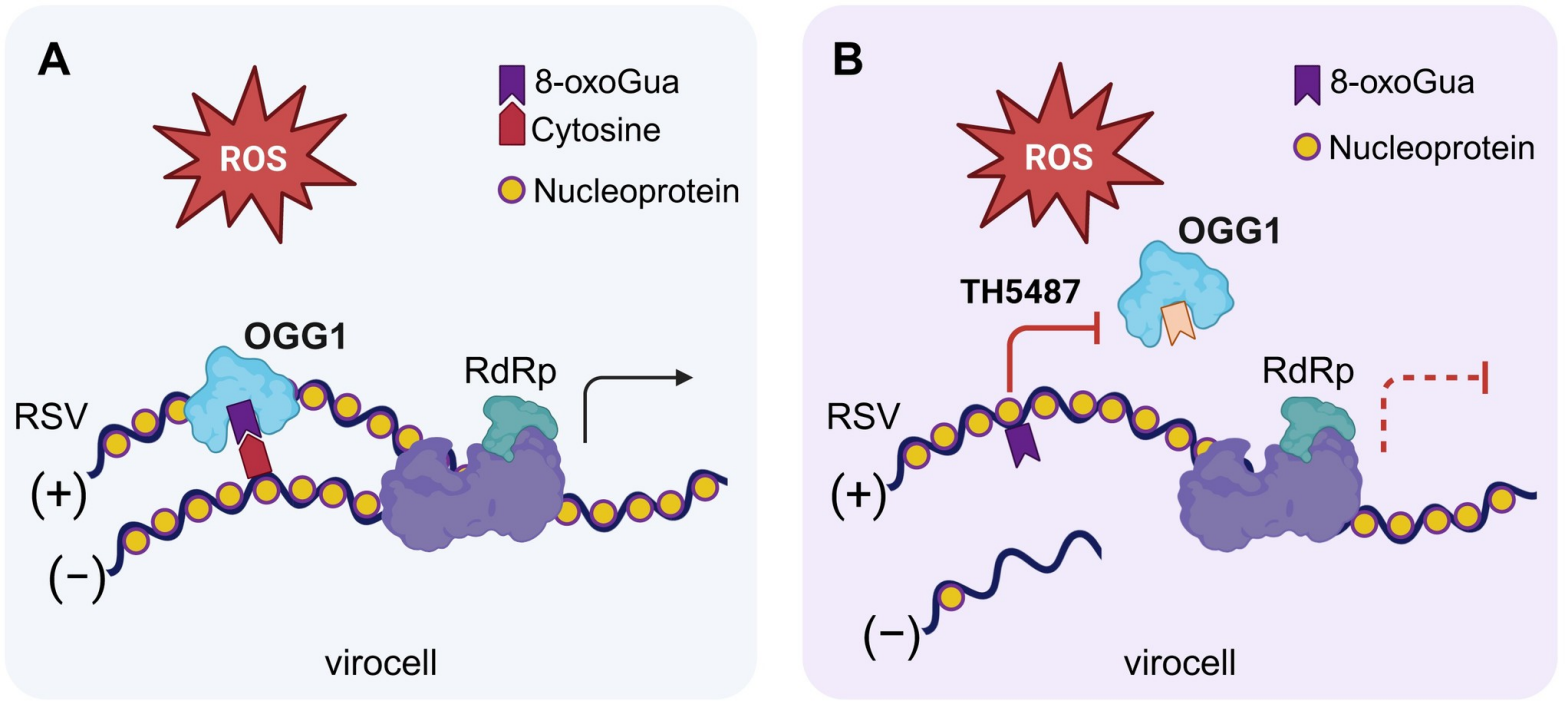
The graphical abstract highlights OGG1’s role in safeguarding viral genome integrity during RSV replication. Elevated ROS levels strategically alter host mechanisms to detect oxidative modifications in viral RNA. (A) OGG1 assumes a pivotal role in this process, co-opted by viral nucleoproteins to facilitate precise base pairing in progeny genomes. (B) Inhibiting OGG1’s ability to detect 8-oxoGua compromises the accuracy of viral replication, resulting in reduced viral progeny production. RdRp, RNA-dependent RNA polymerase. Figure created with Biorender.com.

This study provides the first evidence of oxidative base modification in RNA, specifically 8-oxoGua, identified in RSV N protein-associated RNAs within the oxidative cellular environment. Upon detection of 8-oxoGua in viral RNA, OGG1 is recruited and anchored by the N protein (Fig 8). This mechanism is particularly significant as viruses exploit it to enhance genome fidelity in an oxidizing virocell environment. Loss of OGG1 function markedly lowers viral RNA and protein levels, resulting in decreased viral titers in cultured cells and a mouse infection model. Given the ubiquitous presence of ROS in the life cycles of various pathogenic viruses, these findings expand OGG1’s role beyond its traditional function in maintaining genomic fidelity to safeguarding viral genome stability. Our study paves the way for further exploration, offering an alternative approach to detecting viral RNA that involves base reading rather than the direct cleavage of modified bases.

We have identified 8-oxoGua within RNAs that interact with the N protein extracted from virocells. Oxidative modifications to viral RNA may be explained by a unique nucleotide interaction pattern within the RSV N protein-RNA complex, where each N subunit interacts with seven nucleotides, leaving four bases unprotected [46,47]. This indicates a preferential vulnerability of RNA bases when they lack full protection against oxidative stress. Gua oxidation can occur through direct reactions with reactive species and the migration of charges along the RNA strand towards regions with the lowest redox potential, such as guanine islands [48]. We found low levels of 8-oxoGua in virions that can be explained by further oxidation to guanidinohydantoin or spirohydantoins, as 8-oxoGua serves as an effective ‘positive hole’ sink, playing an essential role in absorbing and neutralizing oxidants, thereby protecting other bases from oxidative injury [49,50]. Since 8-oxoGua accumulation in RNAs has long been considered protective [51], we speculate that the antigenome, with its higher levels of Gua content, shields the genome in progeny virions.

In single-stranded nucleic acids, 8-oxoGua can adopt two alternate conformations: *syn* and *anti*. The *anti*-conformation allows for accurate base pairing with cytosine [52], while the *syn*-conformation causes 8-oxoGua to functionally mimic thymine, pairing with adenine and potentially leading to mutagenesis [53]. This duality suggests a protective mechanism, possibly due to OGG1’s specific affinity for 8-oxoGua paired with cytosine [54,55]. Compared to OGG1 knockout cells, OGG1-expressing cells exhibit significantly increased levels of genomic RNA and yield of infectious progeny. In RNA, unlike in DNA, this interaction does not involve base excision. OGG1’s functional roles in the context of RSV RNA may involve ensuring the *anti*-conformation of 8-oxoGua and precise incorporation of cytosine into the viral genome. Primer extension assays demonstrate that the addition of OGG1 tends to increase the length of polymerase synthesis products (Fig 7J). Therefore, we propose that the interaction of OGG1 with 8-oxoGua in the antigenome has potential implications for the fidelity of RNA synthesis by RNA-dependent RNA polymerase (RdRp) *in vivo*.

Results from hybridization coupled EMSA (Fig 3E) demonstrated the association of OGG1 with specific sequences within the antigenome at the 5’ end of the G gene, while RIP-sequencing confirmed the absence of OGG1 binding sites within the G gene body (Figs 4B and S3). This observation aligns with the higher mutation frequency observed in the G gene among RSV A and B strains [56,57]. In contrast, sequence alignment revealed abundant OGG1 enrichment peaks on the antigenome L gene (Figs 4B and S3), correlating with the relatively low variability of the L gene. OGG1’s role in ensuring accurate replication from the antigenome template, which is susceptible to oxidative guanine modification, underscores its importance in facilitating precise cytosine base pairing in the progeny genome.

While we have demonstrated that OGG1 interacts with the N protein in the absence of viral RNA, this interaction does not necessarily imply that OGG1 exclusively prioritizes either the N protein or viral RNA during the viral life cycle. If OGG1 predominantly interacts with the N protein, which uniformly coats RNA throughout the genome and antigenome, it raises questions about how OGG1 selectively recognizes and acts at specific sites within this context. The requirement for OGG1 to interact with 8-oxoGua, which involves complementary cytosine pairing within the single-stranded RSV genome structure, suggests that the N protein serves as an essential anchor facilitating OGG1’s functional interactions with its substrates. Therefore, the N protein-RNA complex likely presents a more structured substrate for OGG1 to interact with effectively. OGG1’s ability to efficiently recognize 8-oxoGua amidst undamaged bases within high-order chromatin structures [58] suggests its capacity to access the viral nucleocapsid. Investigating OGG1 as a therapeutic target unveils opportunities not only for combating RSV but also for addressing a broader range of viral pathogens, including the Zika virus and DNA viruses such as the African swine fever virus [59,60].

In conclusion, this study has identified a crucial mechanism by which the replication machinery of RSV mitigates the effects of ROS on viral RNAs. The presence of 8-oxoGua, an abundant RNA base modification, poses a significant challenge due to its ambiguous nature and high mutagenic potential. To counteract excessive genome modifications, the virus utilizes the DNA repair protein OGG1. OGG1 seems to play a pivotal role for the virus by recognizing 8-oxoGua primarily in the antigenome, particularly in guanine-rich regions. This interaction ensures accurate base pairing during the synthesis of progeny genomes, thereby preventing mutagenesis that could result from erroneous base pairing (Fig 8). Consequently, OGG1 enhances genome fidelity and sustains the replication fitness of RSV, especially under oxidative stress conditions.

### Limitations of the study

In the current study, we focused primarily on RSV A strain infection. Further research will be necessary to determine whether the replication of other RSV strains or other families of single-stranded, negative-sense RNA viruses are similarly affected by OGG1. Additionally, direct evidence is needed to definitively identify the specific sites of 8-oxoGua in the RSV antigenome from virocells. Further investigation is also required to understand the specific mechanisms of OGG1 in its putative interactions with the RSV antigenome and the regulatory role of the *L* gene in the RSV lifecycle.

## Materials and methods

### Ethics statement

All work involving respiratory syncytial virus, human primary, and established cells was approved by the Institutional Biosafety Committee and performed in the ABSL-2 approved laboratory. Animal experiments were conducted in accordance with the NIH Guide for the Care and Use of Experimental Animals and were approved by the University of Texas Medical Branch Animal Care and Use Committee (Approval no. 0807044D).

### Cell lines

Human Small Airway Epithelial Cells (hSAECs, ATCC PCS-301-010) and HEp-2 (ATCC CCL-23) were acquired from the American Type Culture Collection (Manassas, VA). hSAECs were maintained in Small Airway Epithelial Cell Growth Medium (C-21270, PromoCell). HEp-2 cells were maintained in Dulbecco’s modified Eagle’s medium (DMEM; Life Technologies) supplemented with 5% fetal bovine serum. To create OGG1 knockout hSAECs, CRISPR/Cas9 technology was employed [36]. The genetically modified hSAECs were maintained in Small Airway Epithelial Cell Growth Medium containing 2 μg/mL of puromycin (61-385-RA, Corning) to ensure the survival of successfully edited cells.

### Virus stock and preparation

We used the human RSV A2 strain (ATCC VR-1544), which was propagated using HEp-2 cells. In brief, twenty T150 flasks of HEp-2 cells were infected with RSV at a multiplicity of infection (MOI) of 0.01. After an hour of adsorption, the unbound virus was removed by PBS, and supplemented by fresh culture medium containing 2% fetal bovine serum. Virions were purified on discontinuous sucrose gradients, following previously established methods [61]. Viral titers were determined using the plaque assays as previously described [62]. The purified virus stocks were aliquoted and stored at -80°C until further use.

### Assessment of ROS levels

Kinetic changes in ROS levels were measured using 2’, 7’-dichlorodihydrofluorescein diacetate (H_2_DCF-DA, 35848, Sigma-Aldrich). H_2_DCF-DA, a lipophilic and non-fluorescent compound, easily penetrates the cell membrane and is subsequently deacetylated to form the oxidant-sensitive 2′, 7’-dichlorodihydrofluorescein (H_2_DCF). Upon oxidation, H_2_DCF is converted to the highly fluorescent 2’, 7’-dichlorofluorescein (DCF), which can be quantified to assess ROS levels. For the assay, H_2_DCF-DA was added 3 min before the end of the incubation period, achieving a final concentration of 5 μM. Following incubation, cells were washed and lysed in buffer (50 mM Tris-HCl, pH 7.5, 150 mM NaCl, 1 mM EDTA, 1 mM EGTA, 1% NP-40), and the lysate was clarified by centrifugation. The fluorescence intensity of DCF was immediately measured using a Synergy H1 Hybrid Multi-Mode Reader (BioTek) with excitation/emission wavelengths of 485 nm/535 nm.

For the detection of ROS in infected cells *in situ*, CellROX Green (C10444, Invitrogen) was employed according to the manufacturer’s instructions. Briefly, CellROX Green was added at various times post-infection and incubated for 30 min. After incubation, cells were washed three times with PBS to remove excess dye. Selected fields were then imaged using an ECHO Hybrid Microscope System (ECHO BICO Company) equipped with a built-in digital camera.

### Dot-Blot

For the detection of oxidized proteins, whole cell lysates were prepared in serial dilutions and applied to a Bio-Dot Apparatus (1706545, Bio-Rad) for blotting on a PVDF membrane. Nitrotyrosine, a marker of protein nitration, was detected using an anti-Nitrotyrosine antibody (06–284, Millipore-Sigma). RNA dot-blot was performed with modifications to a method previously described [29]. Total RNA was extracted and spotted a positively charged nitrocellulose membrane, following by immunoblotting with anti-8-oxoGua antibody 15A3 (12501, QED Bioscience).

### Quantification of 8-oxoGua levels using liquid chromatography-tandem mass spectrometry (LC_MS/MS)

RSV RNA from virions was extracted using an RNeasy Mini kit (Qiagen). In the context of RSV-infected hSAECs (MOI = 1, 24 hpi), cells underwent lysis using RIPA buffer, followed by treatment with RNase A (20 μg/mL) and RNase T1 (5U) at 37°C for 30 min to digest naked viral RNA. After a brief centrifugation, the cell lysate was incubated with a Biotin-labeled RSV antibody (7950–0104, Bio-Rad), allowing for the specific capture of RSV ribonucleoprotein complexes. These complexes were then isolated using Streptavidin beads (11206D, Invitrogen) and purified using TRIzol reagent to ensure the extraction of viral RNA interacting with N protein. RNA (50 ng) from both virions and virocells was digested by Nuclease P1 (M0660S, NEB) at 37°C for 15 min, and then adding alkaline phosphatase (M0371S, NEB) at 37°C for 30 min. Reaction mixes were dried down under nitrogen gas before resuspending in 30 μL of 80% of 0.1% formic acid/20% acetonitrile. Ten microliters of the resuspended samples were injected for LCMS analysis. In addition to the samples, standards were prepared between 0.4–200 ng/mL. 25 μL of each standard was dried down, resuspended, and analyzed in the same manner as the samples. LC_MS/MS—was performed on an Acquity Premier UHPLC (Waters) coupled to a QTRAP 6500 mass spectrometer (SCIEX). Modified nucleosides were separated via reversed phase chromatography on an xSelect Premier HSS T3 column (Waters). Mobile phases included: A) 0.1% formic acid in water, B) 0.1% formic acid in acetonitrile. LCMS peak areas were integrated using Skyline (v23.1.0.380, MacCoss Software). Peaks were chosen that most closely matched the standards. Integrated peak areas were normalized to the total ng of RNA used in the digestion reactions using custom R scripts (v4.3.2; Rstudio, v2023.12.0) to give “Peak Area / ng RNA”. Similarly, the ratio of 8-oxoguanine to guanine was calculated to give a relative ratio value.

### Electrophoretic mobility shift assay (EMSA)

EMSA was performed following a previously described method [29], using Cy5-labeled RNA oligonucleotides. The secondary structure of the undenatured single-stranded probe was predicted using "RNA structure" software [63]. For preparation of the double-stranded probe, complementary RNA strands were heat-denatured and then annealed by slow cooling to eliminate any secondary structure. For hybridization-coupled EMSA, total RNA isolated from RSV-infected cells (MOI = 1) and harvested at 24 hpi, resuspended in deionized water to achieve a concentration of approximately 1 μg/μL. One μg of RNA was mixed with a 20 nM Cy5-labeled probe in hybridization buffer (100 mM Tris-HCl, pH 8; 50 mM NaCl; 1 mM EDTA), denatured at 95°C for 5 min, and then allowed to hybridize overnight. Mung bean nuclease (M0250S, New England Biolabs) was utilized to digest any single-stranded, unhybridized probes and RNAs at 30°C for 30 min. Hybrids containing 8-oxoGua were identified by adding 100 nM of OGG1 (ENZ-253, ProSpec) and incubating for 30 min on ice. The resulting protein-DNA complexes were separated on a 6% DNA retardation gel (EC6365BOX, Invitrogen) using 0.5× TBE buffer and visualized using an Amersham Imager 680 (Global Life Sci. Solutions, Marlborough, MA).

In orthogonal approach of performing EMSA with OGG1 mutant, we transfected expressing vector to OGG1 knockout hSAECs. Expressing vector of FLAG-tagged wild-type OGG1 (wtOGG1), the phenylalanine 319 alanine mutant OGG1 (F319A), or the lysine 249 glycine mutant (K249Q) were characterized in our previous publication [18]. After 48 h transfection, whole cell lysates were prepared and quantified by BCA protein assay kit (23225, Thermo Scientific).

### Oligonucleotide excision assay

To assess the excision activity of OGG1, RNA and DNA oligonucleotides containing 8-oxoGua (Integrated DNA Technologies, Coralville, IO) were used (sequences listed in S2 Table). Briefly, 20 nM Cy5-labeled probes were incubated with OGG1 (ENZ-253, ProSpec) in assay buffer (10 mM HEPES, pH 7.9, 10 mM KCl, 1.5 mM MgCl_2_, 1 mM DTT). After a 15-minute incubation at room temperature, the reaction was stopped by adding 10 μL loading buffer (containing 8 μL of formamide and 2 μL of 10 mM of NaOH) and heating for 5 min at 95°C to denature the samples. The cleaved products were separated from intact probes in a 15% TBE-Urea Gel (EC68852BOX, Invitrogen). Subsequently, the gel was visualized using an Amersham Imager 680.

### RNA-immunoprecipitation (RIP) and RIP-Seq

To ensure the purity of our RNA samples and avoid DNA contamination, samples were treated with RNase free DNase I (M0303, New England Biolabs). To further refine our RNA samples, we performed an enrichment of polyadenylated RNA (polyA+ RNA) from the total RNA using Oligo dT beads, which were used specifically for qRT-PCR analysis. This enrichment was accomplished using one or two rounds of the Magnetic mRNA Isolation Kit (S1550, New England Biolabs).

To perform the RNA-immunoprecipitation (RIP) assay, we used the RNA ChIP-IT Immunoprecipitation Kit (53024, Active Motif), incorporating specific modifications to meet our experimental requirements. hSAECs were infected with the RSV (MOI = 1). At 24 hpi, the virocells were crosslinked using 1% methanol-free formaldehyde for 10 min at room temperature. The crosslinking reaction was terminated by adding glycine to a final concentration of 0.125 M. After washing the cells three times with ice-cold PBS-EDTA, we collected them through centrifugation (3500 rpm for 10 min at 4°C). Cells lysis was performed in cold RIPA buffer supplemented with 100 μM phenylmethylsulfonyl fluoride (PMSF) and a proteinase inhibitors cocktail. Following brief sonication, we centrifuged the cell lysates at 16,000 × *g* for 10 min. During the preparation process, we employed the iron chelator deferoxamine mesylate (252750, Millipore-Sigma) to prevent additional oxidation. The supernatant was treated with RNase to remove viral RNAs that are not packaged by nucleoprotein. We then subjected the supernatant to IP with an anti-OGG1 antibody (PA5-86046, Invitrogen) or anti-nucleoprotein (ab94806, Abcam) for 4 hours, using head-over-tail rotation for effective mixing. We incubated the ribonucleoprotein-antibody complexes with magnetic beads for 1 hour at 4°C and subsequently washed them five times with RIPA buffer. The crosslinks were reversed by adding 2 μL of 5M NaCl and 2 μL of Proteinase K to each sample, followed by incubation at 42°C for 1 hour to digest the proteins. The samples were then incubated at 65°C for 1.5 hours to reverse the crosslinks. Following this, RNA was extracted using TRIzol, according to the instruction manual. Libraries were sequenced on an Illumina MiniSeq sequencer at UTMB NextGen Sequencing Core Facility (Director: Dr. H. Hao) with the paired-end 75-bp setting. Barcode sequences were trimmed from the sequencing reads and PCR duplicates were removed. Processed reads were aligned to the human hg38 genome (GenBank GCA_000001405.29) and RSV (GenBank: M74568.1) virus sequence with STAR/2.7.11a. Peaks enriched for reads mapping to RSV RNA were analyzed with deeptools/3.2.0. Peaks enriched for reads mapping to cellular RNAs were annotated with the MACS3 program [64]. Raw data have been deposited at the Gene Expression Omnibus (GEO) repository: GSE269769.

### Co-immunoprecipitation (Co-IP)

Flag-OGG1 expressing hSAECs were either infected with RSV (MOI = 1) or mock-infected for 24 hours. The cells were lysed using RIPA buffer supplemented with cOmplete Protease Inhibitors (Roche), PhosSTOP Phosphatase Inhibitor Cocktail (Roche), 1 mM DTT, and 1 mM PMSF. Following brief sonication, the lysate was clarified by centrifugation at 14,000 × *g* for 10 min. Protein concentrations were determined, and 1 mg of protein was used for IP with anti-OGG1 (PA5-86046, Invitrogen), anti-FLAG (F1804, Millipore-Sigma) or a control IgG (Santa Cruz) for 4 hours at 4°C. Protein A/G Mix Magnetic Beads were then added and incubated for 1 hour with rotation. After five washes in RIPA buffer, proteins were eluted by boiling in 2x Laemmli sample buffer and subjected to SDS-PAGE.

### Protein sample digestion for LC_MS/MS

Protein A/G mix magnetic beads with bound proteins from Co-IPs described above were rinsed twice with 50 μL of 50 mM Triethylammonium bicarbonate buffer (TEAB), pH 7.1. Proteins were eluted by adding 50 μL of 5% SDS, 50 mM TEAB, pH 7.1 and incubated at 37°C for 30 min. Samples were further prepared as described [65]. Briefly, the sample was reduced by adjusting the solution to 10 mM TCEP (Thermo, #77720) and incubation at 65°C for 30 min. The sample was then cooled to room temperature, alkylated with 1 μL of 500 mM iodoacetamide, and allowed to react for 30 min at room temperature in the dark. 2.7 μL of 12% phosphoric acid is added to the 54.7 μL protein solution. 165 μL of binding buffer (90% Methanol, 100 mM TEAB pH 8.5) was then added to the solution. The resulting solution was then added to an S-Trap spin column (protifi.com) and passed through the column using a bench top centrifuge (60s spin at 1,000 × *g*). The spin column was washed with 150 μL of binding buffer and centrifuged. This was repeated two times. 30 μL of 20 ng/μL Trypsin was added to the protein mixture in 50 mM TEAB pH 8.5 and incubated at 37°C overnight. Peptides were eluted twice with 75 μL of 50% acetonitrile, 0.1% formic acid. Aliquots of 20 μL of eluted peptides were quantified using the Quantitative Fluorometric Peptide Assay (Pierce, Thermo Fisher Scientific). An eluted volume of peptides corresponding to 4.2 μg of peptides was dried in a speed vac and resuspended in 20 μL 1.67% acetonitrile, 0.08% formic acid, 0.83% acetic acid, 97.42% water and placed in an auto sampler vial.

### LC_MS/MS analysis

Peptide mixtures were analyzed by nanoflow LC_MS/MS using a nano-LC chromatography system (UltiMate 3000 RSLCnano, Dionex), coupled on-line to a Thermo Orbitrap Fusion mass spectrometer (Thermo Fisher Scientific, San Jose, CA) through a nanospray ion source (Thermo Scientific). A trap and elute method were applied. The trap column was a C18 PepMap100 (100 μm × 2 cm, 5 μm particle size) from Thermo Scientific. The analytical column was an Acclaim PepMap 100 (75 μm × 25 cm) from Thermo Scientific. After equilibrating the column in 98% solvent A (0.1% formic acid in water) and 2% solvent B (0.1% formic acid in acetonitrile (ACN)), 2 μL of sample was injected onto the trap column and subsequently eluted (300 nL/min) by gradient elution from the C18 column as follows: isocratic at 2% B, 0–5 min; 2% to 4% B, 5–6 min; 4% to 25% B, 6–59 min; 25% to 44% B, 59–64 min; 44% to 90% B, 64–66 min; isocratic at 90% B for 1 min, 90% to 5% B, for 1 min; isocratic at 5% B for 1 min (increase flow to 600 nL/min); 5% to 90% B from 69–71 min; isocratic at 90% B for 2 min; 90% to 2% B, for 1 min; isocratic at 2% B, 74–86 min; isocratic at 2% B for 1 min (reduce flow to 450 nL/min); isocratic at 2% B for 2 min (reduce flow to 300 nL/min); and isocratic at 2% B till 90 min.

All LC_MS/MS data were acquired using XCalibur, version 4.4 (Thermo Fisher Scientific) in positive ion mode using a data-dependent acquisition (DDA) method with a 3 sec cycle time. The survey scans (m/z 375–2000) were acquired in the Orbitrap at 120,000 resolutions (at m/z = 400) in profile mode, with a maximum injection time of 50 msec and an AGC target of 400,000 ions. The S-lens RF level was set to 60. Isolation was performed in the quadrupole with a 1.6 Da isolation window, and HCD MS/MS acquisition was performed in centroid mode with detection in the Orbitrap at 30,000 resolutions, with the following settings: parent threshold = 25,000; normalized collision energy = 32%; maximum injection time 35 msec; AGC target 20,000 ions. Monoisotopic precursor selection (MIPS) and charge state filtering were on, with charge states 2–10 included. Dynamic exclusion was used to remove selected precursor ions, with a +/- 10 ppm mass tolerance, for 30 sec after acquisition of one MS/MS spectrum. LC_MS/MS analysis was performed in the Mass Spectrometry Core Facility (Director: W. Russell) of University of Texas Medical Branch at Galveston.

### Database searching

Tandem mass spectra were extracted and charge state deconvoluted by Proteome Discoverer (Thermo Fisher, version 2.5.0.402). Deisotoping is not performed. All MS/MS spectra were searched against Uniprot database of reviewed Respiratory Syncytial Virus sequences, human (obtained June 11th, 2019), and common laboratory contaminants (cRAP, thegpm.org) using Sequest. Searches were performed with a parent ion tolerance of 10 ppm and a fragment ion tolerance of 0.6 Da. Trypsin is specified as the enzyme, allowing for three missed cleavages. Fixed modification of Carbamidomethyl (C) and variable modifications of deamidation (NQ) and oxidation (M) were specified in Sequest. Protein and peptide FDR was set to 1% and protein identification required 2 peptides per protein. The mass spectrometry proteomics data have been deposited to the ProteomeXchange Consortium via the PRIDE [66] partner repository with the dataset identifier PXD053262 and 10.6019/PXD053262.

### Pull-down assays

*In vitro* pull-down assays were performed in accordance with methodologies previously outlined [27]. For these experiments, Pierce Glutathione Agarose (16100, Thermo Scientific) and Dynabeads (10103D, Invitrogen) were utilized following the recommendations provided by their manufacturers. Specifically, GST-tagged OGG1 (TP720109, OriGene, Rockville, MD) was immobilized onto Glutathione Agarose, and subsequently incubated with varying concentrations of His-tagged nucleoprotein (NP-424V, Creative BioMart, Shirley, NY), or His-tagged M2-1 protein (purified locally). Concurrently, His-tagged nucleoprotein was attached to Dynabeads and exposed to increasing concentrations of GST-OGG1. The mixtures were then rotated at room temperature for 10 min, after which the beads underwent four washes with Binding/Wash Buffer in preparation for SDS-PAGE analysis. Post-electrophoresis, membranes were subjected to immunoblotting using specific antibodies targeting OGG1 and the RSV nucleoprotein, thereby facilitating the identification of these proteins within the complexes retrieved from the pull-down assays.

### Purification of RSV M2-1 protein

An optimized sequence of the RSV M2-1 gene fused with an N-terminal Histidine (His) tag was cloned into the vector pET28a (pET28a-His-M2-1) and confirmed by sequencing. His-tagged M2-1 was produced as previously described [67]. Briefly, pET28a-His-M2-1 transfected *E*. *coli* [BL21 (DE3) Star (ThermoFisher)] were cultured in LB medium supplemented with kanamycin (50 μg/mL) until reaching an absorbance of 0.7 at 600 nm. Induction of protein expression was achieved by adding IPTG (0.5 mM) and ZnSO_4_ (50 μM), followed by incubation at 37°C for 3 h. The harvested cells were subsequently frozen in liquid nitrogen and stored at –80°C. For purification, the cells were thawed and resuspended in lysis buffer (50 mM Tris-HCl, pH 7.4, 300 mM NaCl, 1 mM β-mercaptoethanol) supplemented with protease inhibitor cocktail. Cell disruption was performed using a microfluidizer (Microfluidics, Newton, MA) at 15,000 PSI for three cycles. The lysate was clarified by centrifugation at 18,000 rpm for 45 min at 4°C, followed by filtration through a 0.22-μm filter (Millipore, Burlington, MA). The clarified lysate was loaded onto a 5 mL nickel HisTrap HP column (GE Healthcare, Piscataway, NJ) equilibrated with a fast-flow buffer. The column was washed with 20 mM imidazole, followed by a high salt buffer (1 M NaCl). The eluted fractions containing His-M2-1 were pooled and further purified using a cation-exchange Hi-Trap SP column (GE Healthcare, Piscataway, NJ) to remove nucleic acids. The purity of His-M2-1 protein in the final elution buffer (20 mM Tris-HCl, pH 7.4, 150 mM NaCl) was confirmed by measuring the absorbance ratio at 260 nm/280 nm and Western blot analysis.

### Immunofluorescence and proximity ligation assay (PLA)

Cells growing on microscope coverslips were washed with PBS, air dried, and fixed in acetone-methanol (1:1) for 20 min at -20°C. After complete drying, cells were rehydrated in PBS for 15 min and then blocked with 1% BSA for 60 min at room temperature. Primary antibodies were applied at dilutions recommended by the manufacturers in PBST and incubated overnight at 4°C. The primary antibodies used included anti-OGG1 (NB100-106, Novus), anti-nucleoprotein (ab94806, Abcam), anti-Nitrotyrosine (06–284, Millipore-Sigma), and anti-M2-1 (ab94805, Abcam). For secondary detection, antibodies conjugated to Alexa Fluor 488 or Alexa Fluor 594 (Molecular Probes) were used. Nuclei were counterstained with 4’, 6-diamidino-2-phenylindole (DAPI) at a concentration of 10 ng/mL. Pearson’s correlation coefficient for fluorophores was calculated using Image J v1.51 software [68].

For the proximity ligation assay (PLA), the Duolink PLA kit (LNK-92101-KI01, OLink Bioscience) was used according to the manufacturer’s instructions. Fixed cells were incubated overnight at 4°C with primary antibodies against OGG1 and the N protein, washed with 1 × buffer A (DUO82049, Millipore Sigma), and subsequently incubated with secondary antibodies conjugated with MINUS and PLUS probes for 1 h at 37°C. After washing with 1 × buffer A, the ligation mix was added for 30 min at 37°C, followed by amplification on each sample for 100 min at 37°C. Finally, cells were washed with buffer B (DUO82049, Millipore Sigma) and mounted with mounting medium.

### Primer extension

Extension efficiency assays were performed following the methods outlined previously [53]. To prepare the DNA primer/RNA template duplexes, 10 nM of a DNA primer (5’Cy5-TGCGTTGGTCCTT-3’) and 10 nM of an RNA template (5’-GGG/r8-oxoGua/CAAAUGCAAACAUGUCCAAAAACAAGGACCAACGCA-3’) were combined and subjected to an annealing process: 95°C for 5 min, 55°C for 5 min, then gradually brought to room temperature. The primer extension reaction was carried out in a 20 μL volume using a modified Moloney Murine Leukemia Virus Reverse Transcriptase (2680B, TaKaRa), following the manufacturer’s protocol. To prepare for electrophoresis, an equal volume of 2× gel loading buffer was added to the reaction mixture, which was then heated to 95°C for 10 min. The samples were subsequently analyzed on a 15% TBE-Urea Gel (EC68852BOX, Invitrogen) and the results were visualized using an Amersham Imager 680 (Global Life Sci. Solutions, Marlborough, MA).

### siRNA and transfection

SMARTpool siRNAs targeting OGG1 (L-005147-00-0010), and non-targeting control siRNAs were acquired from Dharmacon (Lafayette, CO). The SMARTpool siRNA product comprises four distinct siRNA sequences, supplied as a single, pooled reagent to enhance the efficiency and specificity of gene silencing. All siRNA transfections were conducted using Lipofectamine RNAiMAX Reagent (Invitrogen), following the manufacturer’s recommended protocol.

### Crystal violet staining

To assess the cytopathic effect induced by RSV infection, the cells were stained with crystal violet [69]. The infected samples were washed with PBS and stained with 0.01% crystal violet in 20% methanol for 10 min. After staining, the samples were rinsed with deionized water. The stained samples were then imaged using an Amersham Imager 680. For quantification, the crystal violet stain was solubilized by adding 200 μL of methanol to each sample followed by incubation for 20 min. The optical density (OD) of the solubilized stain was then measured at 570 nm using a plate reader (BioTek Synergy H1, BioTek Instruments Inc., Winooski, VT).

### Lactate dehydrogenase (LDH)-cytotoxicity assay

RSV-infected hSAECs (MOI = 1) were treated with either vehicle (solution without inhibitor) or TH5487 (4-(4-Bromo-2-oxo-3H-benzimidazol-1-yl)-N-(4-iodophenyl)piperidine-1-carboxamide), TH2840 (4-(2-oxo-2,3-dihydro-1H-1,3-benzodiazol-1-yl)-N-phenylpiperidine-1-carboxamide), or O8 (3,4-Dichlorobenzo[b]thiophene-2-carbohydrazide) following virus inoculation. After 24 hpi, cell culture medium was collected and subjected to a colorimetric LDH assay using the LDH Cytotoxicity Assay Kit II (K313-500, BioVision) following the manufacturer’s instructions. The absorbance of the medium supernatants was measured at 450 nm using a microplate reader (Synergy H1 Hybrid Multi-Mode Reader; BioTek).

### Animal challenge and treatment

Ten-week-old BALB/c mice (The Jackson Laboratory, Bar Harbor, ME, USA) housed in the animal research facility of the UTMB were used for these studies. Under mild isoflurane anesthesia, randomly selected groups of mice (50% female and 50% male) were challenged via intranasal route with RSV (10^6 PFU per mice) in 60 μL of phosphate-buffered saline solution (pH 7.4). TH5487 (SelleckChem; 30 mg/ kg) in 200 μL of solvent (5% DMSO, 10% Tween 80 in saline) was administered via the peritoneal route every 8 h after RSV challenge. All experiments were performed in accordance with the NIH Guide for Care and Use of Experimental Animals and approved by the University of Texas Medical Branch Animal Care and Use Committee (Approval no. 0807044D).

### Statistical analysis

To evaluate the data, we initially performed an unpaired one-way analysis of variance (ANOVA) across all data sets. This approach allowed us to determine if there were any statistically significant differences among the various groups under investigation. Following the ANOVA, we further analyzed significant differences between specific conditions using Student’s *t* test. We set a threshold where a *p*-value of less than 0.05 was considered to indicate statistical significance.

## Supporting information

S1 Fig RSV N protein is in the RSV-antibody pull-down from virocells. hSAECs were mock-infected or RSV-infected (MOI = 1) for 24 h. Whole cell lysates were prepared using RIPA buffer and incubated with RNase A (20 μg/mL) and RNase T1 (5U) at 37°C for 30 min to digest naked RNA. Immunoprecipitation (IP) was performed using an anti-RSV antibody (7950–0104, Bio-Rad) or control IgG, followed by SDS polyacrylamide gel electrophoresis (PAGE). The blotted proteins were then probed with an anti-N antibody (ab94806, Abcam). Anti-Actin immunoblotting from whole cell lysates served as a loading control. IB, immunoblot.
(TIF)

S2 Fig Intact active-site pocket required for OGG1 binding to RNA. Parallel cultures of OGG1 knockout cells were transfected with vectors expressing FLAG-tagged wild-type OGG1 (wtOGG1), the phenylalanine 319 alanine mutant OGG1 (F319A), or the lysine 249 glycine mutant OGG1 (K249Q). After 48 h of transfection, whole cell lysates (WCL) were prepared. Following protein quantification, 2 μg of WCL was used as the starting point, and two-fold serial dilutions were performed to incubate with RNA probes (8oxo-rG: rC oligonucleotides), followed by EMSA experiments.
(TIF)

S3 Fig Integrative genome viewer (IGV) screenshot showing OGG1 RIP-Seq signals. hSAECs were RSV infected (MOI = 1) for 24 h, and RNA was isolated as outlined in Materials and Methods. The data are represented as log2 of the ratio of reads obtained from anti-OGG1 IP compared to input RNA, mapped against the RSV genome (GenBank: M74568.1). Upper panel: Forward alignment of OGG1 RIP-Seq signals to the reference, corresponding to the RSV antigenome. The positions of the guanine-rich sequences 5’-GGGGCAAAT, 5’-GGGACAAAAT, GGGG, GGG, and GG are indicated. Lower panel: Reverse alignment of OGG1 RIP-Seq signals to the reference, corresponding to the RSV genome. The positions of the sequences 5’-ATTTGCCCC, 5’-ATTTTGTCCC, CCCC, CCC, and CC are indicated. RIP, RNA immunoprecipitation; Rep1, Rep2, experimental replicates.
(TIF)

S4 Fig Gene Ontology (GO) graphs depicting pathway clusters identified within the OGG1 interactome in RSV-infected cells. (TIF)

S5 Fig Physical interactions between OGG1 and M2-1 in virocells and in vitro. (A) Immuno-staining of OGG1 and M2-1 in virocells (MOI = 1). The co-localization measurement between fluorophores (Alexa 594 vs Alexa 488) was assessed using the intensity of individual fluorophore pixels, calculated as the Pearson correlation coefficient (R). Scale bars: 20 μm. (B-C) GST pull-down assays demonstrating the interaction between OGG1 and the M2-1 protein. GST-tagged OGG1 (50 nM) was incubated with increasing concentrations of His-tagged M2-1 and subsequently immunoblotted using an anti-M2-1 antibody (B). In a reciprocal setup, His-tagged M2-1 protein (50 nM) was incubated with varying concentrations of GST-OGG1, followed by immunoblotting with an anti-OGG1 antibody (C). M2-1, transcriptional processivity and antitermination factor; GST, glutathione S-transferase.
(TIF)

S6 Fig Disruption of OGG1-RNA interaction reduces RSV progeny production. (A) OGG1 depletion, but not NEIL2 or MTH1, reduces RSV progeny yield. Left panel: Supernatant fluids collected from RSV-infected hSAECs were analyzed by plaque assays to determine viral titers (MOI = 1, 24 hpi). Right panel: Expression levels of OGG1, NEIL2, and MTH1 in non-targeting (NT) and targeted siRNA-transfected hSAECs were measured by qRT-PCR. n = 3 biological replicates. (B) OGG1 inhibitor TH5487 decreases N and M2-1 protein levels. Western blot analysis of whole cell extracts (MOI = 1, 24 hpi) probed with anti-N and anti-M2-1 antibodies. (C) TH5487 reduces RSV progeny yield. Tenfold serial dilutions of virocell supernatants were added to triplicate HEp-2 cell. Cells were formalin-fixed (0.5%), stained with crystal violet, and photographed at 5 days post-infection. (D) Quantification of crystal violet staining was performed by measuring optical density (OD) at 570 nm. Cells infected with undiluted and 100-fold diluted supernatant was compared. n = 3 biological replicates. Statistical analysis was conducted using an unpaired Student’s *t* test, with results shown as means ± SD. Significance levels are indicated as **p < 0.01, ***p < 0.001.
(TIF)

S7 Fig TH5487 decreases RSV-induced release of lactose dehydrogenase (LDH). hSAECs were treated with indicated concentrations of TH5487, O8, or TH2840 post RSV inoculum (MOI = 1). Cell culture medium was harvested at 24 hpi to perform LDH assay according to the manufacturer’s instruction. The absorbance at 450 nm was determined using a microplate reader (Synergy H1 Hybrid Multi-Mode Reader; BioTek). n = 3 biological replicates. Statistical analysis was conducted using an unpaired Student’s *t* test, with results shown as means ± SD. Significance levels are indicated as ***p<0.001. TH5487, 4-(4-Bromo-2-oxo-3H-benzimidazol-1-yl)-N-(4- iodophenyl) piperidine-1-carboxamide; O8, 3, 4-Dichlorobenzo[b]thiophene-2-carbohydrazide; TH2840, 4-(2-oxo-2, 3-dihydro-1H-1, 3-benzodiazol-1-yl)-N- phenylpiperidine-1-carboxamide).
(TIF)

S8 Fig TH5487 decreases RSV replication in lungs. Groups of mice (n = 8) were challenged with purified RSV (10^6 PFU per mouse) via the intranasal route. RSV. TH5487 (30 mg/kg) was administered intraperitoneally at time 0 and every 8 h after RSV challenge. Lung tissues were collected at indicated times after infection. (A) RSV yield was titrated on HEp-2 cells using a plaque assay. (B-C) Total RNA was extracted from lungs, and mRNA was isolated using Oligo dT. After mRNA selection, genomic RNA was quantified. Levels of RSV genome (B) and *G* mRNA (C) were determined by qRT-PCR. n = 8 from 3 biological replicates. Statistical analysis was conducted using an unpaired Student’s *t* test, with results shown as means ± SD. Significance levels are indicated as *p<0.05, ***p<0.001.
(TIF)

S1 Table Primer sequences for RT q-PCR. (TIF)

S2 Table Oligo sequences for EMSA and excision assay. (TIF)

S3 Table LC_MS/MS analysis for OGG1 interactomes. (XLSX)
