# Supplementary figures and images for: 8-Oxoguanine DNA Glycosylase1 conceals oxidized guanine in nucleoprotein-associated RNA of respiratory syncytial virus
Source: PLoS Pathog. 2024 Oct 16;20(10):e1012616. doi: 10.1371/journal.ppat.1012616 (PMC11515973; doi:10.1371/journal.ppat.1012616)

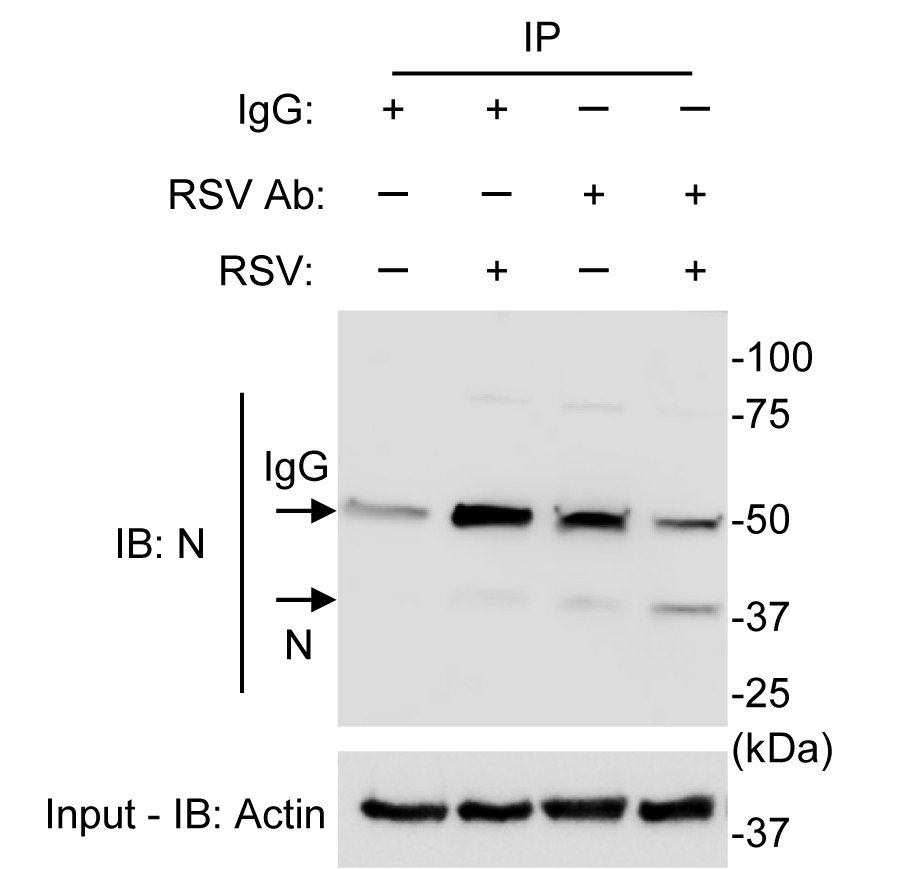

Supplement: S1 Fig — hSAECs were mock-infected or RSV-infected (MOI = 1) for 24 h. Whole cell lysates were prepared using RIPA buffer and incubated with RNase A (20 μg/mL) and RNase T1 (5U) at 37°C for 30 min to digest naked RNA. Immunoprecipitation (IP) was performed using an anti-RSV antibody (7950–0104, Bio-Rad) or control IgG, followed by SDS polyacrylamide gel electrophoresis (PAGE). The blotted proteins were then probed with an anti-N antibody (ab94806, Abcam). Anti-Actin immunoblotting from whole cell lysates served as a loading control. IB, immunoblot. (TIF) [file ppat.1012616.s001.tif]

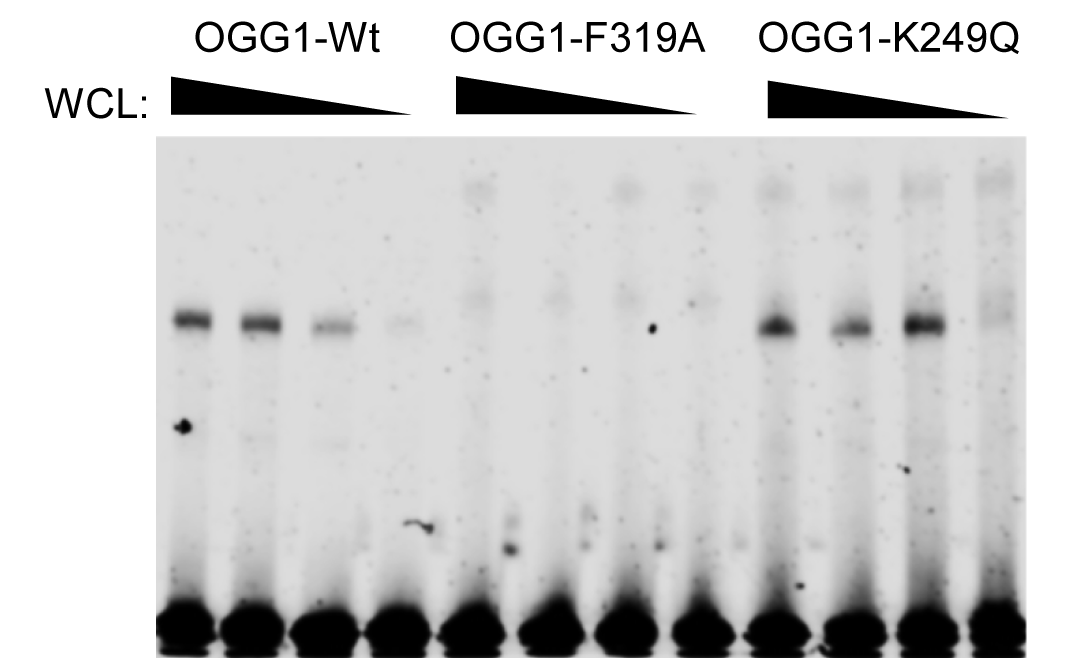

Supplement: S2 Fig — Parallel cultures of OGG1 knockout cells were transfected with vectors expressing FLAG-tagged wild-type OGG1 (wtOGG1), the phenylalanine 319 alanine mutant OGG1 (F319A), or the lysine 249 glycine mutant OGG1 (K249Q). After 48 h of transfection, whole cell lysates (WCL) were prepared. Following protein quantification, 2 μg of WCL was used as the starting point, and two-fold serial dilutions were performed to incubate with RNA probes (8oxo-rG: rC oligonucleotides), followed by EMSA experiments. (TIF) [file ppat.1012616.s002.tif]

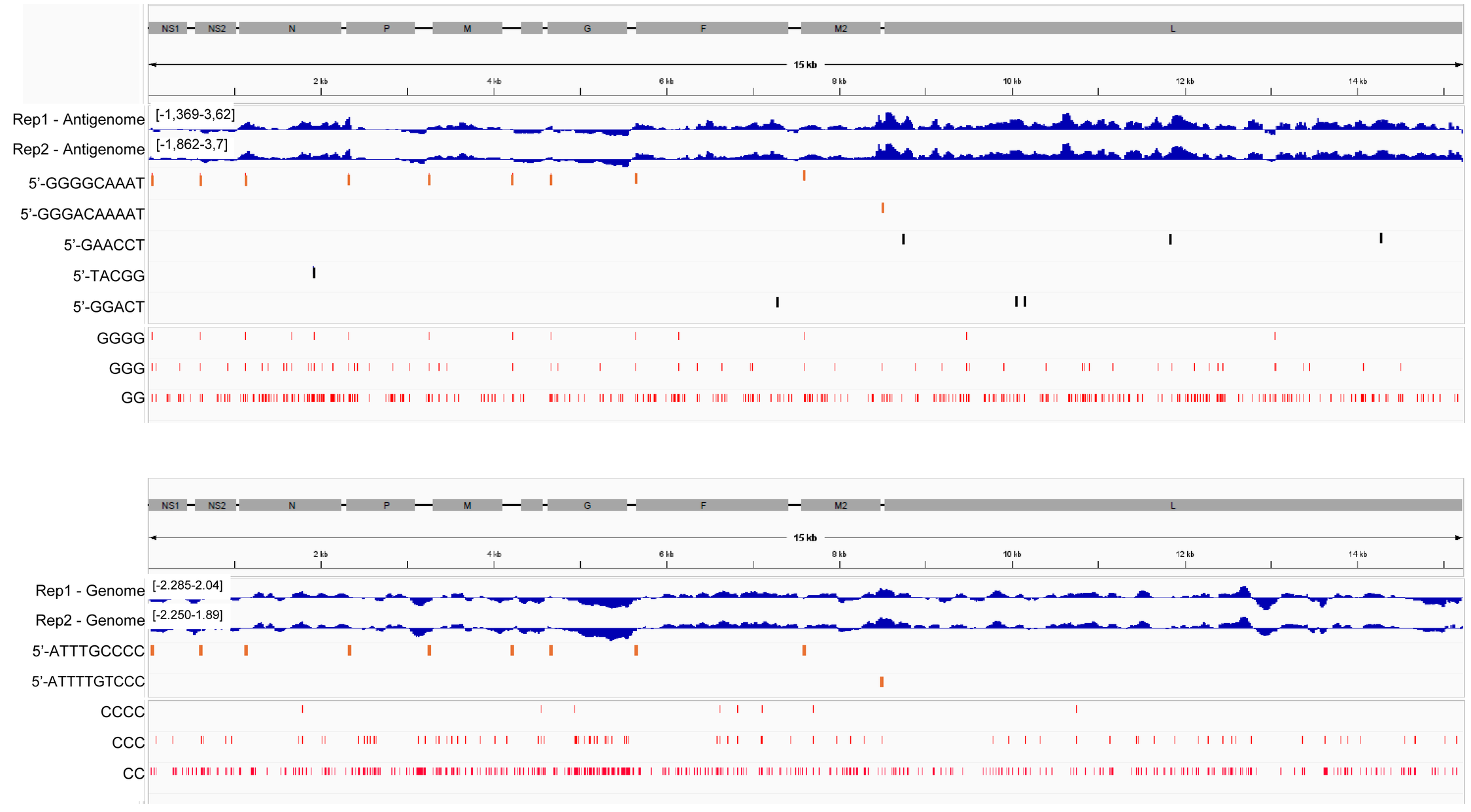

Supplement: S3 Fig — hSAECs were RSV infected (MOI = 1) for 24 h, and RNA was isolated as outlined in Materials and Methods. The data are represented as log2 of the ratio of reads obtained from anti-OGG1 IP compared to input RNA, mapped against the RSV genome (GenBank: M74568.1). Upper panel: Forward alignment of OGG1 RIP-Seq signals to the reference, corresponding to the RSV antigenome. The positions of the guanine-rich sequences 5’-GGGGCAAAT, 5’-GGGACAAAAT, GGGG, GGG, and GG are indicated. Lower panel: Reverse alignment of OGG1 RIP-Seq signals to the reference, corresponding to the RSV genome. The positions of the sequences 5’-ATTTGCCCC, 5’-ATTTTGTCCC, CCCC, CCC, and CC are indicated. RIP, RNA immunoprecipitation; Rep1, Rep2, experimental replicates. (TIF) [file ppat.1012616.s003.tif]

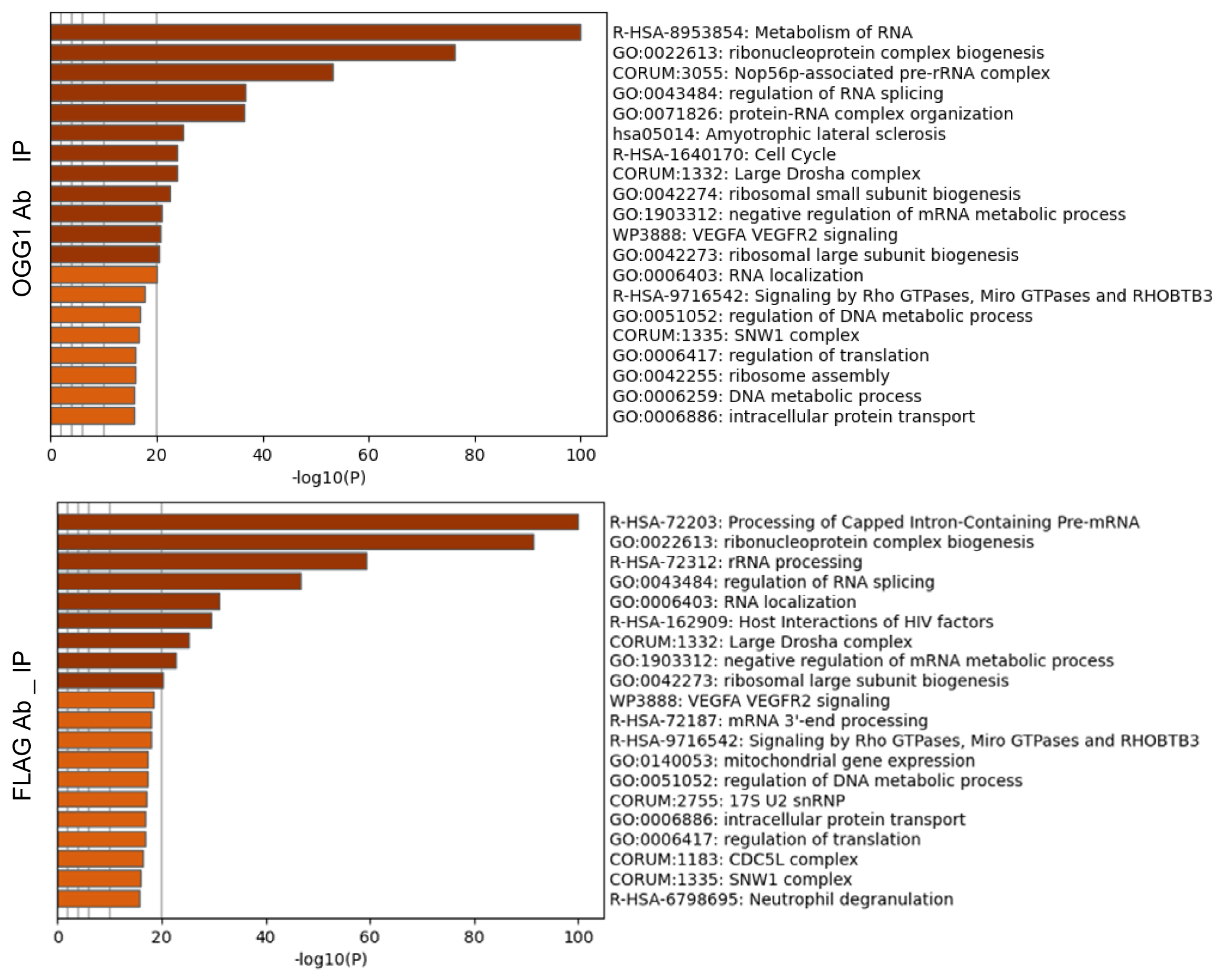

Supplement: S4 Fig — (TIF) [file ppat.1012616.s004.tif]

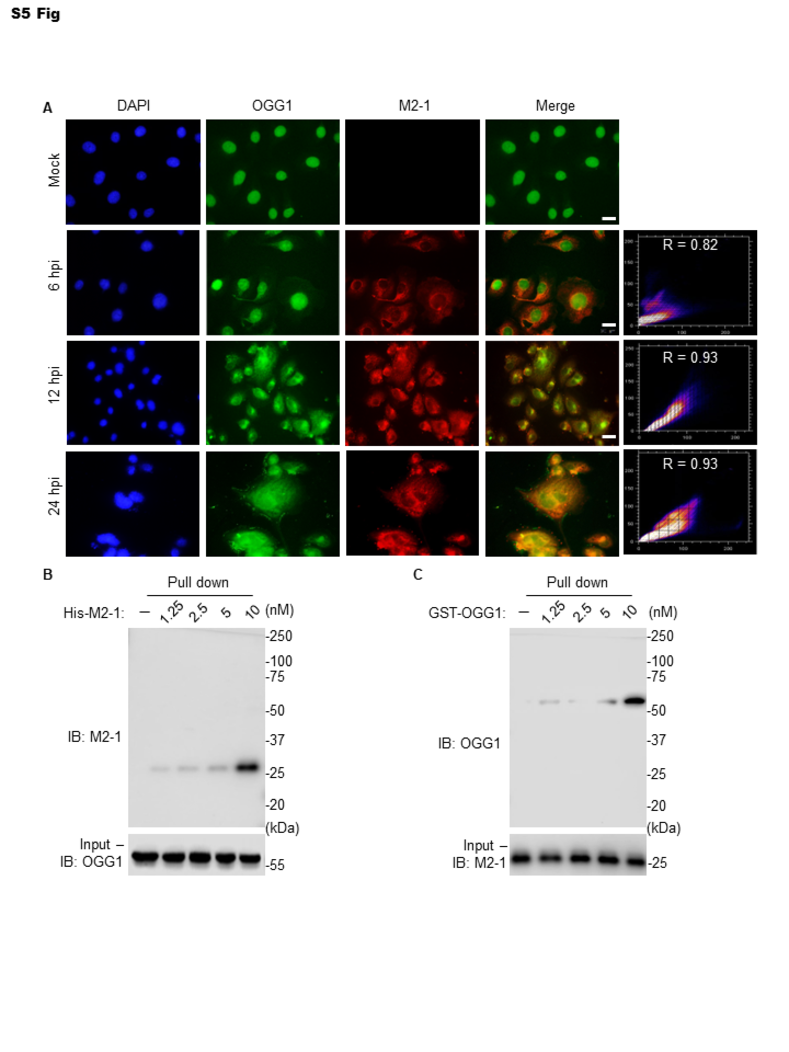

Supplement: S5 Fig — (A) Immuno-staining of OGG1 and M2-1 in virocells (MOI = 1). The co-localization measurement between fluorophores (Alexa 594 vs Alexa 488) was assessed using the intensity of individual fluorophore pixels, calculated as the Pearson correlation coefficient (R). Scale bars: 20 μm. (B-C) GST pull-down assays demonstrating the interaction between OGG1 and the M2-1 protein. GST-tagged OGG1 (50 nM) was incubated with increasing concentrations of His-tagged M2-1 and subsequently immunoblotted using an anti-M2-1 antibody (B). In a reciprocal setup, His-tagged M2-1 protein (50 nM) was incubated with varying concentrations of GST-OGG1, followed by immunoblotting with an anti-OGG1 antibody (C). M2-1, transcriptional processivity and antitermination factor; GST, glutathione S-transferase. (TIF) [file ppat.1012616.s005.tif]

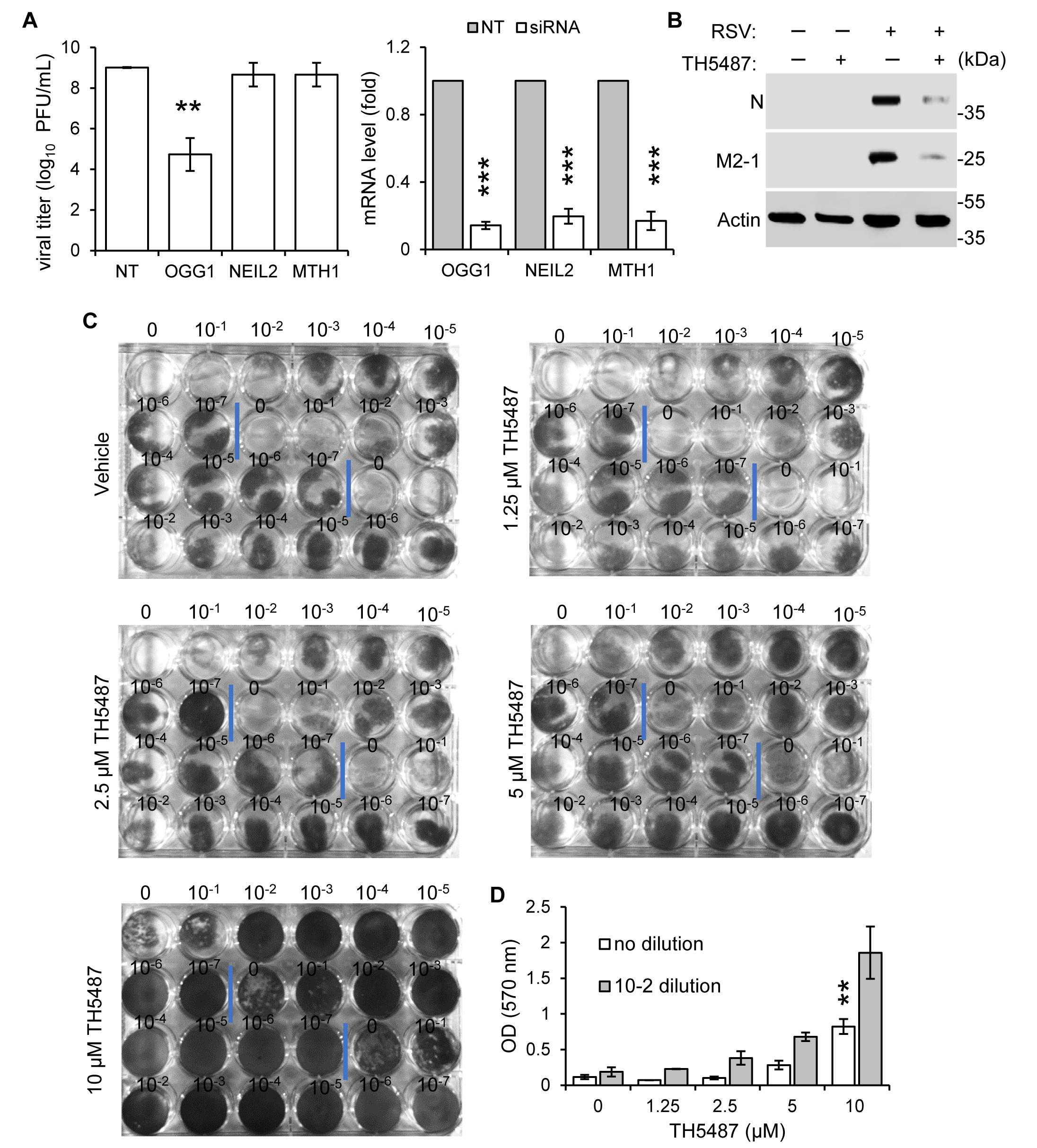

Supplement: S6 Fig — (A) OGG1 depletion, but not NEIL2 or MTH1, reduces RSV progeny yield. Left panel: Supernatant fluids collected from RSV-infected hSAECs were analyzed by plaque assays to determine viral titers (MOI = 1, 24 hpi). Right panel: Expression levels of OGG1, NEIL2, and MTH1 in non-targeting (NT) and targeted siRNA-transfected hSAECs were measured by qRT-PCR. n = 3 biological replicates. (B) OGG1 inhibitor TH5487 decreases N and M2-1 protein levels. Western blot analysis of whole cell extracts (MOI = 1, 24 hpi) probed with anti-N and anti-M2-1 antibodies. (C) TH5487 reduces RSV progeny yield. Tenfold serial dilutions of virocell supernatants were added to triplicate HEp-2 cell. Cells were formalin-fixed (0.5%), stained with crystal violet, and photographed at 5 days post-infection. (D) Quantification of crystal violet staining was performed by measuring optical density (OD) at 570 nm. Cells infected with undiluted and 100-fold diluted supernatant was compared. n = 3 biological replicates. Statistical analysis was conducted using an unpaired Student’s t test, with results shown as means ± SD. Significance levels are indicated as **p < 0.01, ***p < 0.001. (TIF) [file ppat.1012616.s006.tif]

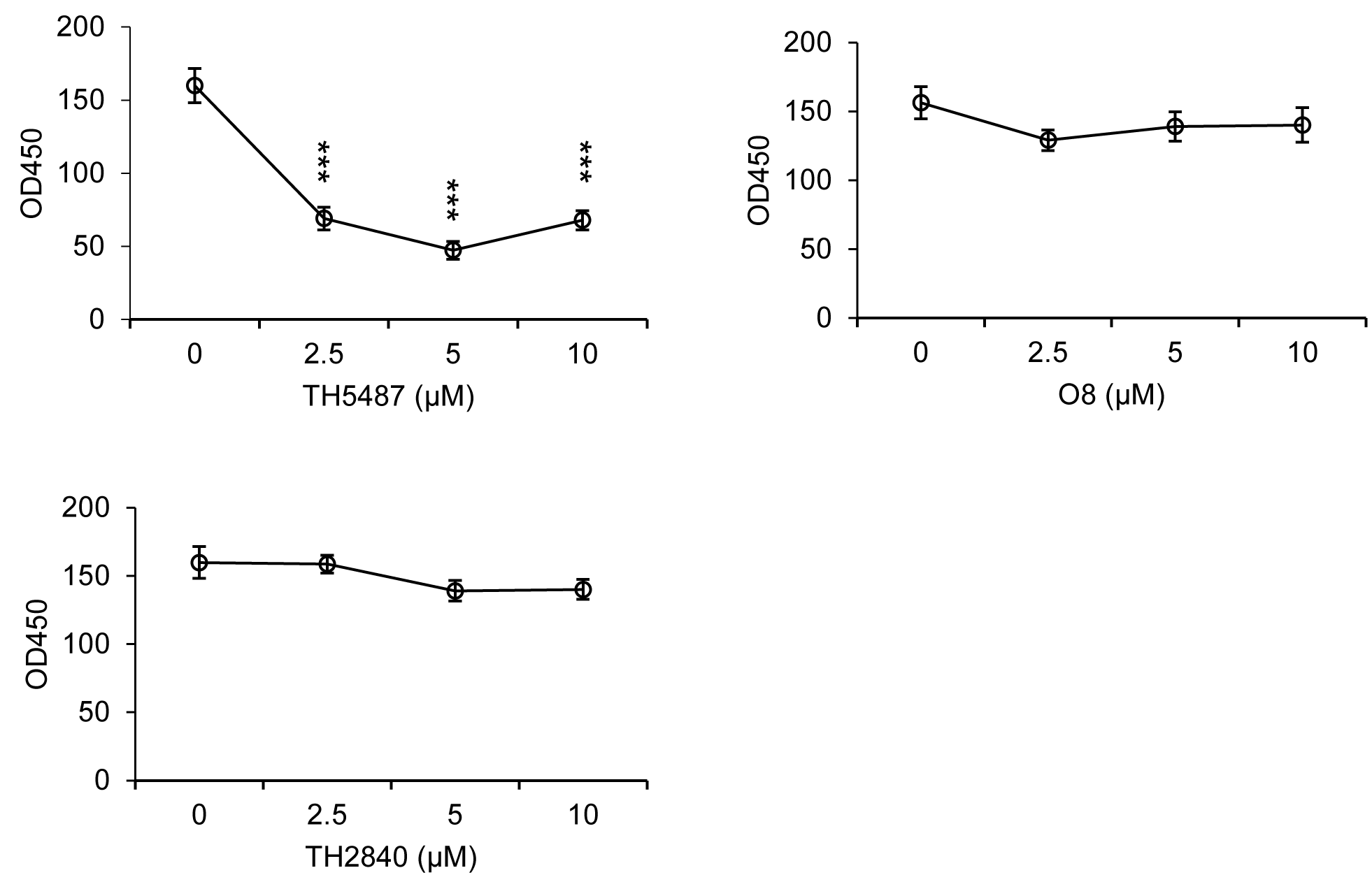

Supplement: S7 Fig — hSAECs were treated with indicated concentrations of TH5487, O8, or TH2840 post RSV inoculum (MOI = 1). Cell culture medium was harvested at 24 hpi to perform LDH assay according to the manufacturer’s instruction. The absorbance at 450 nm was determined using a microplate reader (Synergy H1 Hybrid Multi-Mode Reader; BioTek). n = 3 biological replicates. Statistical analysis was conducted using an unpaired Student’s t test, with results shown as means ± SD. Significance levels are indicated as ***p<0.001. TH5487, 4-(4-Bromo-2-oxo-3H-benzimidazol-1-yl)-N-(4- iodophenyl) piperidine-1-carboxamide; O8, 3, 4-Dichlorobenzo[b]thiophene-2-carbohydrazide; TH2840, 4-(2-oxo-2, 3-dihydro-1H-1, 3-benzodiazol-1-yl)-N- phenylpiperidine-1-carboxamide). (TIF) [file ppat.1012616.s007.tif]

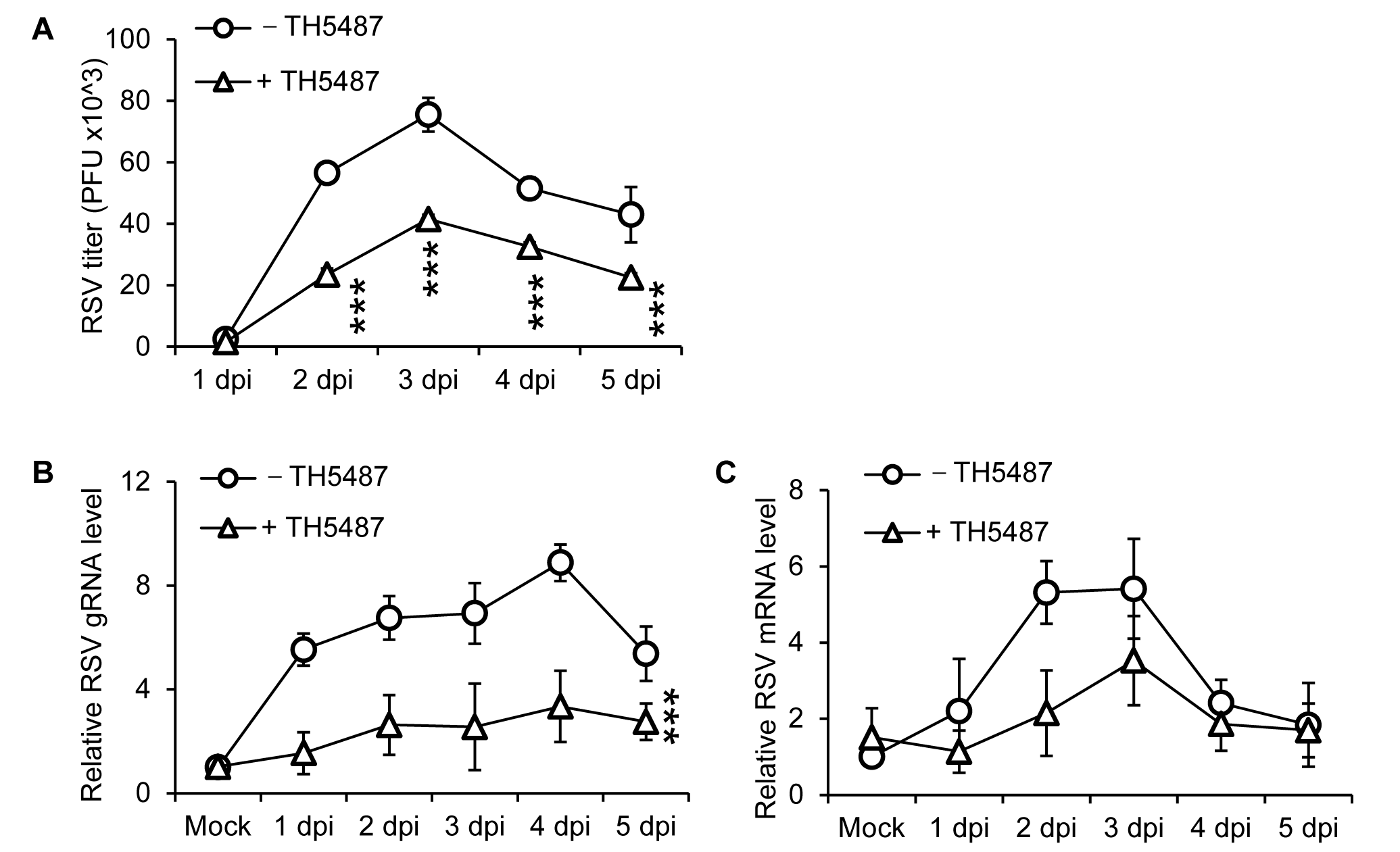

Supplement: S8 Fig — Groups of mice (n = 8) were challenged with purified RSV (10^6 PFU per mouse) via the intranasal route. RSV. TH5487 (30 mg/kg) was administered intraperitoneally at time 0 and every 8 h after RSV challenge. Lung tissues were collected at indicated times after infection. (A) RSV yield was titrated on HEp-2 cells using a plaque assay. (B-C) Total RNA was extracted from lungs, and mRNA was isolated using Oligo dT. After mRNA selection, genomic RNA was quantified. Levels of RSV genome (B) and G mRNA (C) were determined by qRT-PCR. n = 8 from 3 biological replicates. Statistical analysis was conducted using an unpaired Student’s t test, with results shown as means ± SD. Significance levels are indicated as *p<0.05, ***p<0.001. (TIF) [file ppat.1012616.s008.tif]

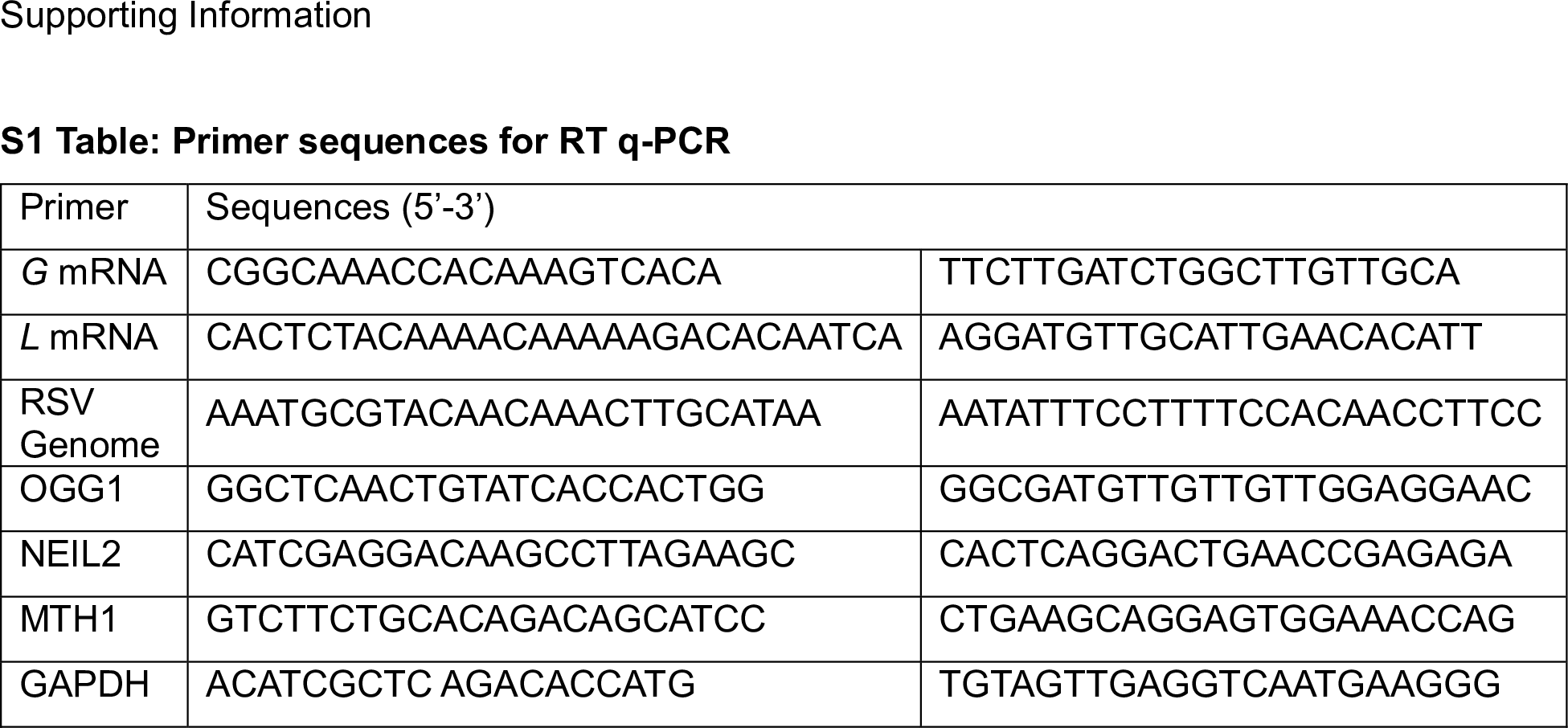

Supplement: S1 Table — (TIF) [file ppat.1012616.s009.tif]

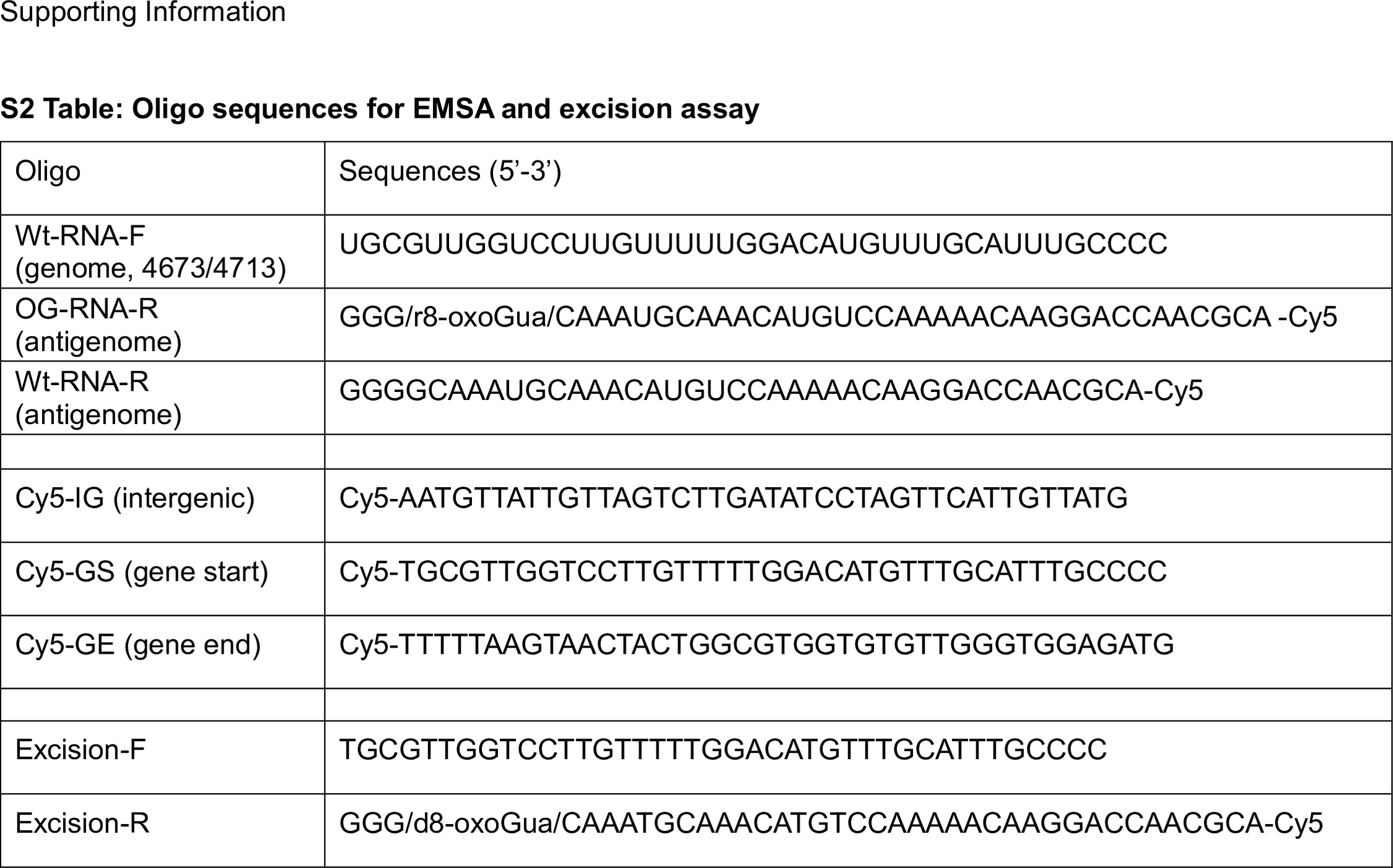

Supplement: S2 Table — (TIF) [file ppat.1012616.s010.tif]
